# Local neuropeptide signaling modulates serotonergic transmission to shape the temporal organization of *C*. *elegans* egg-laying behavior

**DOI:** 10.1371/journal.pgen.1006697

**Published:** 2017-04-06

**Authors:** Navonil Banerjee, Raja Bhattacharya, Michael Gorczyca, Kevin M. Collins, Michael M. Francis

**Affiliations:** 1 Department of Neurobiology, University of Massachusetts Medical School, Worcester, MA United States of America; 2 Department of Biology, University of Miami, Coral Gables, FL United States of America; Katholieke Universiteit Leuven, BELGIUM

## Abstract

Animal behaviors are often composed of distinct alternating behavioral states. Neuromodulatory signals are thought to be critical for establishing stable behavioral states and for orchestrating transitions between them. However, we have only a limited understanding of how neuromodulatory systems act *in vivo* to alter circuit performance and shape behavior. To address these questions, we have investigated neuromodulatory signaling in the context of *Caenorhabditis elegans* egg-laying. Egg-laying activity cycles between discrete states–short bursts of egg deposition (active phases) that alternate with prolonged quiescent periods (inactive phases). Here using genetic, pharmacological and optogenetic approaches for cell-specific activation and inhibition, we show that a group of neurosecretory cells (uv1) located in close spatial proximity to the egg-laying neuromusculature direct the temporal organization of egg-laying by prolonging the duration of inactive phases. We demonstrate that the modulatory effects of the uv1 cells are mediated by peptides encoded by the *nlp-7* and *flp-11* genes that act locally to inhibit circuit activity, primarily by inhibiting vesicular release of serotonin from HSN motor neurons. This peptidergic inhibition is achieved, at least in part, by reducing synaptic vesicle abundance in the HSN motor neurons. By linking the *in vivo* actions of specific neuropeptide signaling systems with the generation of stable behavioral outcomes, our study reveals how cycles of neuromodulation emanating from non-neuronal cells can fundamentally shape the organization of a behavioral program.

## Author summary

Animals have robust mechanisms in place to shape their behavior in a manner that is beneficial both for their survival and for the survival of their progeny. A class of signaling molecules known as neuropeptides have been implicated in driving transitions between behavioral states but we have only a limited understanding of how neuropeptide signaling modulates neural circuit activity *in vivo* to elicit alternate behavioral outcomes. Egg-laying behavior in the model system *C*. *elegans* cycles between clusters of egg-laying and prolonged inactive periods. This temporal organization provides for spatial dispersal of eggs, presumably benefiting progeny by limiting local overcrowding and competition for food. Here we uncover a novel neuromodulatory mechanism that shapes the timing of egg-laying behavior. Specifically, we find that neuromodulatory signaling from a group of non-neuronal cells specifies transitions between active and inactive phases of egg-laying by regulating neurotransmitter release from the motor output neurons of the egg-laying circuit. Advancing our knowledge of how neuropeptides and other modulators act in the context of the circuits in which they are endogenously released will be critical in ongoing efforts to understand how alternate behavioral states, for example those underlying mood or arousal, are encoded.

## Introduction

Animals have robust mechanisms in place to shape their behavior in a manner that is beneficial both for their survival and for the survival of their progeny. In many cases behaviors are composed from discrete, opposing behavioral states. Examples of behaviors that are organized in this manner range from the motor programs underlying feeding or locomotion to more complex behaviors such as mood or arousal [1–5]. In each of these cases, animals adapt their behavior in response to either changes in environmental conditions or internal physiological signals by modifying the duration of component behavioral states.

How the nervous system establishes the duration of particular behavioral states and executes transitions between them, however, is largely unclear. Neuromodulators such as neuropeptides are attractive candidates for orchestrating these processes [6–11]. Pharmacological, biochemical, and electrophysiological studies have shed light on how neuropeptide signaling can modify the activity of neural circuits [12–17]. Neuropeptide signals typically activate or refine patterns of neural activity that are generated from the actions of fast-acting transmitters by altering cellular excitability or by modifying the efficiency of synaptic communication between neurons. However, precisely linking *in vivo* activation of specific neuromodulatory systems with changes in circuit performance and the generation of alternate behavioral outcomes remains a challenge. Uncovering basic principles by which neuromodulators influence circuit activity and behavior in more simple systems provides an important framework for tackling similar questions in the mammalian brain. In particular, the neural circuit controlling egg-laying behavior in the nematode *Caenorhabditis elegans* provides a relatively simple and experimentally tractable system to address these questions.

**Fig 1:**
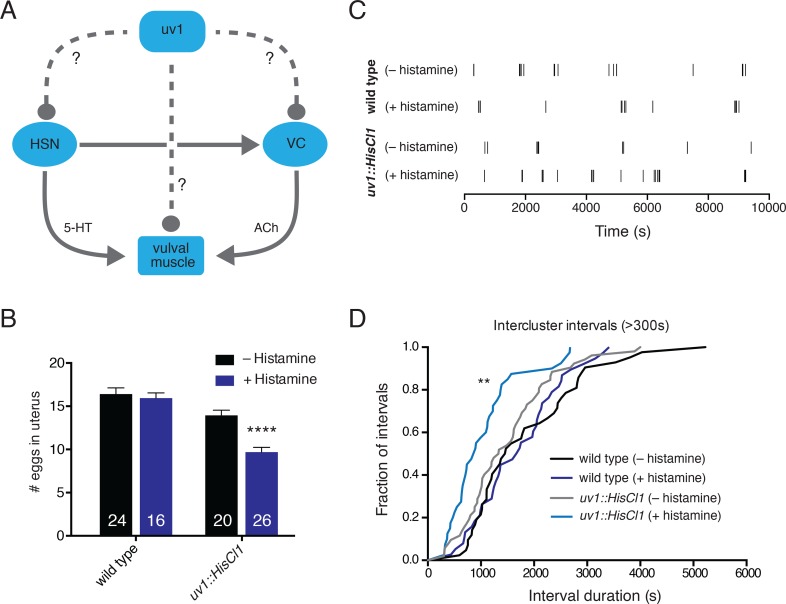
Reducing the activity of neurosecretory uv1 cells increases egg-laying. (A) Schematic diagram of the egg-laying circuit. The HSNs make primarily serotonergic synaptic connections onto Vm2 vulval muscles and the VC5 motor neurons. Cholinergic VC4 and VC5 motor neurons make synaptic connections onto the vulval muscles. Neurosecretory uv1 cells express several neuromodulators but do not have direct postsynaptic targets. Circles represent putative inhibitory connections while arrows denote excitatory connections. Solid lines depict known synaptic connections while dashed lines depict putative extrasynaptic signaling. Connections based on [20,21,46,63]. (B) Quantification of eggs *in utero* for wild type and transgenic animals (*ufIs160*) stably expressing the histamine-gated chloride channel in uv1 cells (*uv1*::*HisCl1*) under control conditions or immediately following exposure (6 hrs) to exogenous histamine (50 mM). *uv1*::*HisCl1* refers to expression of the *Pocr-2*::*HisCl1*::*SL2*::*GFP*::*ocr-2* 3’ UTR transgene. Bars represent mean ± SEM for each condition. Numbers in bars indicate n for each condition. ****p<0.0001, ANOVA with Sidak’s test. (C) Temporal analysis of egg-laying behavior with uv1 silencing. Histamine exposure began 1 hr prior to the start of analysis and continued throughout. Tick marks indicate single egg-laying events. Representative data from one animal is shown for each condition. (D) Cumulative distribution of intercluster intervals between egg-laying events. The frequency of all intervals >300 s is plotted for the conditions indicated. Histamine treatment (light blue) produces a significant leftward shift toward shorter intercluster intervals. WT (–his): n = 42; WT (+ his): n = 38; *uv1*::*HisCl1* (-His): n = 52, *uv1*::*HisCl1* (+His): n = 40. **p<0.01, Mann-Whitney test. n = number of intervals.

Under favorable conditions, *C*. *elegans* egg-laying behavior cycles probabilistically between two alternative states: active phases containing short bursts of egg-laying and prolonged periods of quiescence (inactive phases) [9,18–20]. This temporal organization of egg-laying allows for spatial dispersal of eggs, and may be beneficial for survival of progeny by limiting local overcrowding and competition for food. The core circuitry and signaling machinery controlling egg-laying is well described, but the mechanisms responsible for this temporal organization have remained unclear. Egg-laying occurs when specialized sex-specific muscles contract, opening the vulva and allowing for deposition of eggs. Two serotonergic hermaphrodite-specific neurons (HSN) and two cholinergic ventral C class motor neurons (VC4 and VC5) make excitatory synaptic contacts onto the Vm2 muscles [21]. Egg-laying active states are associated with serotonin release from the HSNs that increases vulval muscle excitability (Fig 1A) [9,20,22–24].

Four neurosecretory cells, known as uv1 cells, are located in close proximity to both HSN processes and the egg-laying musculature, forming a ring through which eggs pass as they exit the uterus and vulva [25]. The uv1 cells do not form synaptic connections with the egg-laying circuitry, but express candidate neuromodulators (e.g. FMRFamide peptides, tyramine), raising the possibility that secretion of neuromodulators from the uv1 cells may allow for temporal coordination of egg-laying activity [26,27]. By linking acute cell-specific activation to alternate behavioral outcomes, we demonstrate that uv1 secretion of neuropeptides encoded by the *nlp-7* and *flp-11* genes inhibits HSN-vulval muscle transmission, setting the duration of inactive phases during egg-laying. Unlike the other neurotransmitter systems described to date involved in the inhibition of egg laying, NLP-7 and FLP-11 peptides do not strictly depend on G_i/o_ G-protein coupled receptor (GPCR) signaling. Instead, inhibition involves redundant functions of G_i/o_ and the *Drosophila* gustatory receptor-like protein EGL-47. Our studies support a model where neuropeptide signaling controls the temporal organization of opposing behavioral states by altering synaptic vesicle abundance in motor output neurons.

## Results

### Altering uv1 activity affects the timing of egg-laying events

To begin to address the role of the uv1 cells in neuromodulatory control of egg-laying, we first asked whether manipulations that alter uv1 activity affect the retention of eggs *in utero*, an indirect measure of egg-laying activity. Wild type adults typically retain about 15 eggs *in utero* under normal laboratory conditions. We used cell-specific expression of a histamine-gated chloride channel (HisCl1) to selectively hyperpolarize the uv1 cells [28]. The *ocr-2* promoter region and 3’ UTR regions provide for selective expression in the uv1 cells and a few additional head and tail neurons [29]. Using these regulatory regions to drive HisCl1 expression, we silenced uv1 cells with exposure to exogenous histamine (6 hrs) and then measured the number of eggs retained in the uterus (Fig 1B). Transgenic animals expressing HisCl1 without exposure to exogenous histamine retain a similar number of eggs to wild type. Histamine-mediated hyperpolarization of the uv1 cells causes a significant reduction in the number of eggs *in utero* (31%, p < 0.0001), consistent with an increased rate of egg-laying. To investigate this further, we next asked whether hyperpolarization of the uv1 cells altered the temporal organization of egg-laying events by monitoring egg-laying in freely moving animals (Fig 1C and 1D). Under favorable growth conditions, bouts of egg-laying (clusters) are separated by prolonged inactive phases (15–60 mins) in which eggs are retained in the uterus. We found that uv1 hyperpolarization causes a significant shift towards shorter inactive phases (Fig 1D).

**Fig 2:**
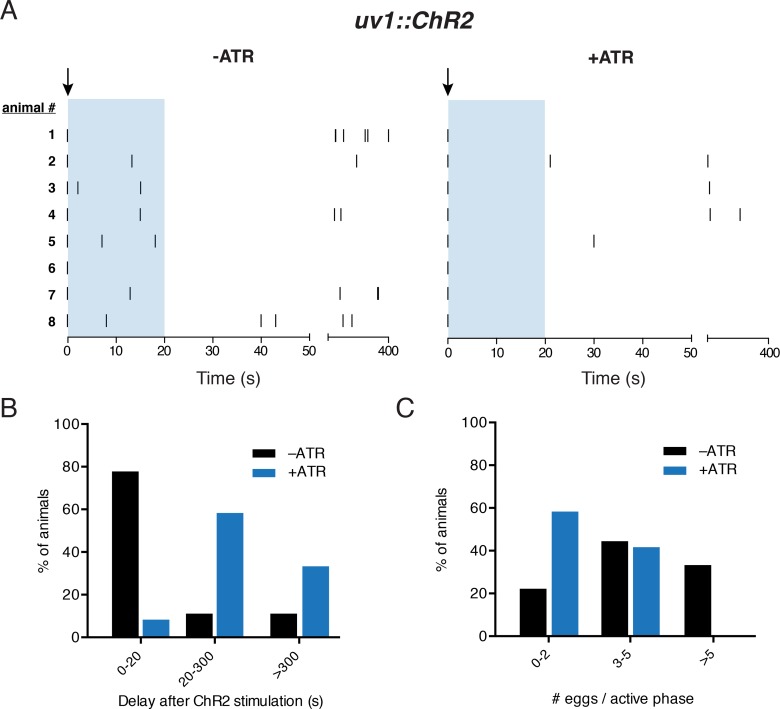
Acute stimulation of uv1 cells inhibits egg-laying activity. (A) Temporal analysis of egg-laying behavior for 400 s immediately following photostimulation of animals stably expressing ChR2 in the uv1 cells (*vsIs189*; *Pocr-2*::*ChR2*::*YFP*::*ocr-2* 3’UTR) under control conditions (-ATR) or with exposure to exogenous retinal (+ATR). Light stimulation (indicated by arrow)(5 s, 100 W/m^2^) was initiated immediately after the first egg-laying event of an active phase. Tick marks indicate single egg-laying events. Representative data from 8 animals for each condition is shown. Blue shading indicates the 0–20 s time interval quantified in Fig 2B (0–20). Two time periods are shown: (1) 50 s immediately following the first egg-laying event (0–50) and (2) the subsequent 350 s (50–400) on a condensed scale. (B) Quantification of time interval between the onset of light stimulus and subsequent egg-laying event. Percent of animals responding within 20 s, 20–300 s, or after >300 s is shown as indicated. -ATR: n = 9, +ATR: n = 12, p<0.0001, Chi-square test. (C) Quantification of total egg-laying events following the initial event within an active phase. Percent of animals laying 0–2, 3–5, or >5 eggs is shown as indicated. -ATR: n = 9, +ATR: n = 12, p<0.0001, Chi-square test.

We next sought to determine whether uv1 activation is sufficient to inhibit egg-laying. To address this question, we expressed channelrhodopsin (ChR2) in uv1 cells using the regulatory regions of *ocr-2* as above. Light stimulation immediately following the initial egg-laying event of an active phase (see Methods) significantly delays subsequent egg-laying events, and also significantly reduces the total number of egg-laying events within an active phase (Fig 2)(S1 Movie). For example, under control conditions a majority (~80%) of animals show a delay between the first and second egg-laying events within an active phase of <20 s (light stimulation, -ATR) (Fig 2B). This proportion is reduced dramatically (to around 10%) when uv1 cells are activated (light stimulation, +ATR). In addition, a significant fraction of uv1 activated animals (~33%, compared with 11% of controls) halt egg-laying for >5 mins following light stimulation, likely indicating termination of the active phase (Fig 2B). Of the animals that progress to a second egg-laying event, the average time interval between the first and second egg-laying events increases from roughly 12 s under control conditions to nearly 38 s with uv1 depolarization. Approximately 30% of the animals tested lay 5 or more eggs within an active phase under control conditions, but active phases with >5 events are not observed with uv1 depolarization (Fig 2C). Moreover, about 60% of uv1-activated animals lay ≤2 eggs after light stimulation compared to just 22% of animals under control conditions. Taken together, our findings provide evidence that uv1-mediated inhibition of egg-laying promotes periods of quiescence in the egg-laying program and plays a key role in setting their duration.

### NLP-7 and FLP-11 neuropeptides are expressed in the uv1 cells and synergistically inhibit egg-laying behavior

**Fig 3:**
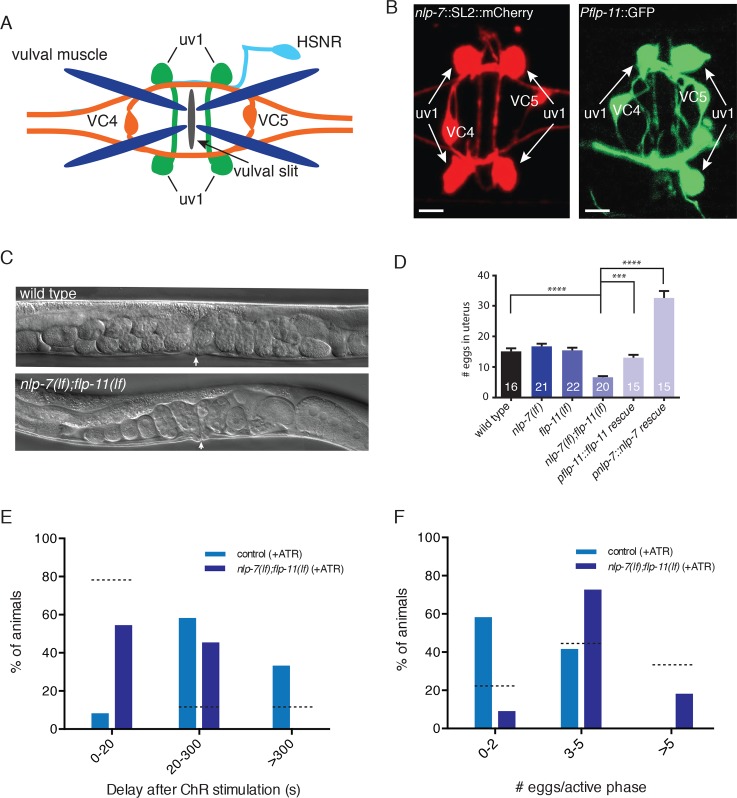
NLP-7 and FLP-11 are expressed in uv1 cells and act synergistically to inhibit egg-laying. (A) Schematic of egg-laying neuromusculature. (B) Fluorescent images of adult hermaphrodites expressing either *Pnlp-7*::NLP-7::SL2::mCherry or *Pflp-11*::GFP [36] reporter transgenes. In addition to the egg-laying circuit, both reporters label head and tail neurons (see S1 and S2 Figs). Scale bars, 5 μm. (C) Representative DIC images of wild type (upper) and *nlp-7(lf);flp-11(lf)* double mutants (lower). Arrows indicate position of the vulva. (D) Quantification of eggs *in utero*. Bars represent mean ± SEM for each genotype. Numbers in bars indicate the n for each group. **** p<0.0001, *** p<0.001 ANOVA with Sidak’s post-hoc test. In this and all subsequent figures, *nlp-7(lf)* and *flp-11(lf)* refer to the *tm2984* and *tm2709* alleles respectively. (E) Time interval between light stimulus and subsequent egg-laying event. For E and F, light stimulation (5 s, 100 W/m^2^) of animals stably expressing *uv1*::*ChR2* was initiated immediately after the first egg-laying event of an active phase. Percent of animals that perform a second egg laying event within 20 s, 20–300 s, or after >300 s is indicated. control, n = 12; *nlp-7;flp-11*, n = 11. p<0.0001, Chi-square test. Dashed lines indicate percent of animals in each category for control stimulation in the absence of retinal (from Fig 2). (F) Quantification of total egg-laying events following the initial event within an active phase. Percent of animals laying 0–2, 3–5, or >5 eggs following uv1 photostimulation is shown. Dashed lines indicated percent of animals in each category for control stimulation in the absence of retinal (from Fig 2). ATR, exogenous retinal. control, n = 12; *nlp-7;flp-11*, n = 11. p<0.0001, Chi-square test.

The dense core vesicle marker IDA-1 is expressed in the uv1 cells, and we noted that an IDA-1::GFP translational fusion produces punctate fluorescence in the uv1 cells, consistent with prior reports documenting the presence of secretory vesicles in uv1 cells [27,30,31] (S1A Fig). These findings suggest that neuromodulatory signals are likely to be important for uv1 regulation of egg-laying. We therefore investigated the expression of several neuropeptide precursors–potential dense core vesicle cargoes–in the uv1 cells. We used a reporter construct in which coding sequence for the fluorescent protein mCherry preceded by the SL2 splice leader was added to the native genomic sequence encoding selected neuropeptide precursors. This bicistronic vector drives expression of the precursor and the mCherry reporter under the control of native regulatory sequences. Amongst the candidates tested, the NLP-7 precursor was of particular interest, producing mCherry fluorescence in cells neighboring the vulva (S1B Fig). We also observed mCherry fluorescence in head and tail neurons, consistent with a prior report [32]. The mCherry fluorescence near the vulva clearly labels the uv1 cells and the VC4 and VC5 motor neurons, and overlaps with that of IDA-1::GFP (Fig 3A and 3B and S1C Fig). The *nlp-7* gene encodes four mature peptides that belong to the SFamide family (S1D Fig), but functions for these peptides have only recently begun to be elucidated [33–35]. We also confirmed expression of a second neuropeptide precursor, *flp-11*, in the uv1 cells using a *Pflp-11*::GFP transcriptional reporter. Consistent with previous reports [36,37], we observed *flp-11*::GFP fluorescence in head and tail neurons, and also prominently in the uv1 cells and VC4 and VC5 motor neurons (S2A Fig and Fig 3A and 3B). While *flp-11*::GFP expression is evident in at least one uv1 cell of all adults tested, and remains detectable throughout adulthood, the number of uv1s labeled is somewhat variable across individuals. The *flp-11* neuropeptide precursor is predicted to give rise to four mature peptides of the FMRFamide class (S2B Fig) and *flp-11* expression in RIS neurons located in the head has been previously implicated in the control of sleep [37]. Our demonstration of *nlp-7* and *flp-11* expression in the egg-laying circuit raises the interesting possibility that local release of these peptides may regulate circuit performance and egg-laying behavior.

To explore the above possibility, we first investigated egg-laying behavior in *nlp-7* and *flp-11* single deletion mutants. *nlp-7(lf)* and *flp-11(lf)* animals were not significantly different from the wild type with respect to the number of eggs retained in the uterus (Fig 3D). Similarly, single deletion of *nlp-7* or *flp-11* does not significantly advance the developmental stage at which embryos are laid (1–8 cell stage: wild type 10%, *nlp-7(lf)* 10%, *flp-11(lf)* 6%, n>80 eggs for each genotype), suggesting that loss of either single neuropeptide precursor does not appreciably affect the rate of egg-laying.

Neuropeptides often work in parallel pathways to mediate specific behavioral outcomes. To test whether NLP-7 and FLP-11 may be acting cooperatively to regulate egg-laying, we investigated egg-laying behavior in *nlp-7(lf);flp-11(lf)* double mutants. Strikingly, we found that the double mutants retain significantly fewer eggs *in utero* than either wild type or the respective single mutants (Fig 3C and 3D). In addition, double mutants lay a significantly higher percentage of eggs in the early cell stage (1–8 cell stage) than wild type (wild type: 10%; *nlp-7(lf);flp-11(lf)*: 58%, n>100 eggs for each genotype)(S3 Fig). Transgenic expression of wild type *nlp-7* or *flp-11* in *nlp-7(lf);flp-11(lf)* double mutants under control of their respective native promoters reverses these effects (Fig 3D). Thus, our findings suggest that either class of peptides, if present at sufficient levels, is capable of slowing the rate of egg-laying. Indeed, transgenic expression of *nlp-7* increases the retention of eggs *in utero* beyond wild type levels, suggesting that a chronic increase in circulating levels of NLP-7 peptides may be sufficient to produce prolonged inhibition of the egg-laying circuit and deficits in egg-laying.

To address whether specific peptide expression in the uv1 cells is sufficient to rescue constitutive egg-laying in *nlp-7(lf);flp-11(lf)* mutants, we expressed either *nlp-7* or *flp-11* under the control of *ocr-2* regulatory sequence. uv1-specific expression of either peptide precursor completely reverses hyperactive egg-laying, supporting the idea that the uv1 cells are an important endogenous source of these peptides for regulation of egg-laying (S3 Fig). We therefore next tested whether uv1-mediated inhibition of egg-laying is dependent on *nlp-7* and *flp-11* neuropeptides by measuring egg-laying responses to uv1 photostimulation in *nlp-7(lf);flp-11(lf)* double mutants (Fig 3E and 3F)(S2 Movie). As described in Fig 2, uv1 stimulation in control animals increases the interval between egg-laying events and reduces the number of events during an active phase. For depolarization of uv1 cells in *nlp-7(lf);flp-11(lf)* double mutants, the interval between consecutive egg-laying events is significantly decreased compared to uv1 stimulation in controls. Moreover, 90% of *nlp-7(lf);flp-11(lf)* double mutants perform ≥3 egg-laying events following light stimulation compared with 40% of controls that perform ≥3 events (Fig 3E and 3F). These results support the idea that secretion of NLP-*7* and FLP-11 peptides in response to stimulation of the uv1 cells is sufficient to inhibit egg-laying. However, we note that the effects of uv1 stimulation are not completely reversed in *nlp-7;flp-11* double mutants, consistent with the possibility that other modulatory signals may also contribute to uv1 regulation of egg-laying.

**Fig 4:**
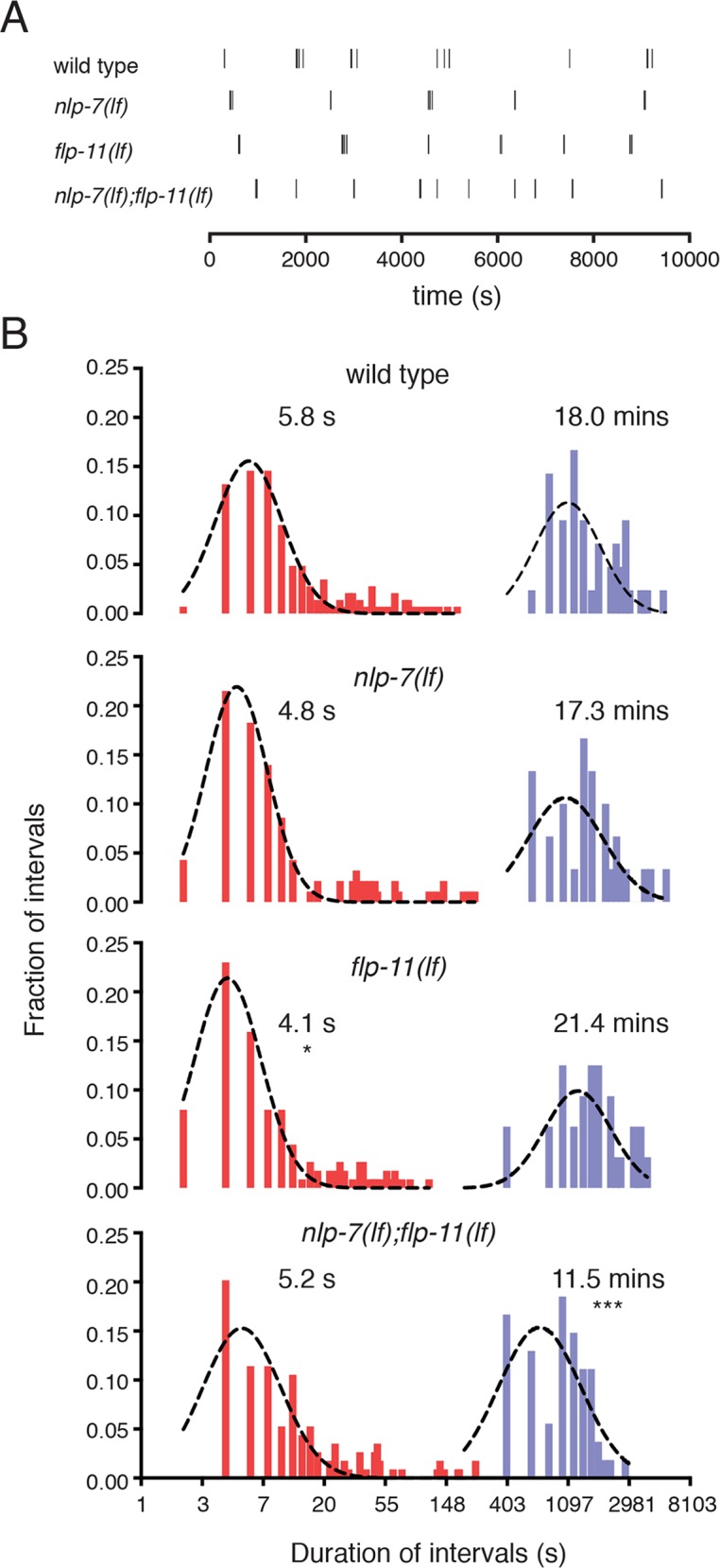
The duration of inactive egg-laying periods is reduced with combined deletion of *nlp-7* and *flp-11*. (A) Temporal analysis of egg-laying behavior. Tick marks indicate single egg-laying events. Representative data from one animal of each genotype is shown. (B) Histograms of intervals between egg-laying events. Intervals are plotted on a natural log scale on the x-axis and their relative frequencies are plotted on the y-axis. Red indicates intracluster intervals (<300 s) and blue indicates intercluster intervals (>300 s). Dashed lines indicate curve fit to Gaussian distribution. Mean intracluster and intercluster intervals for each genotype were calculated from the curve fit (wild type: 5.8 ± 1 s and 18.0 ± 0.02 mins; *nlp-7(lf)*: 4.8 ± 1 s and 17.3 ± 0.02 mins; *flp-11(lf)*: 4.1 ± 1 s and 21.4 ± 0.02 mins; *nlp-7(lf);flp-11(lf)*: 5.2 ± 1 s and 11.5 ± 0.02 mins). wild type: n1 = 144, n2 = 42; *nlp-7(lf)*: n1 = 93, n2 = 30; *flp-11(lf)*: n1 = 113, n2 = 32; *nlp-7(lf);flp-11(lf)*: n1 = 114, n2 = 54. n1 = number of intracluster intervals (<300 s), n2 = number of intercluster intervals (>300 s). ***p<0.001, *p<0.05, Kruskal Wallis test with Dunn’s multiple comparisons test. The average intercluster interval (inactive phase) is significantly shortened in *nlp-7;flp-11* double mutants and egg-laying occurs at earlier developmental stages compared with wild type. These effects are rescued by uv1-specific expression of either *nlp-7* or *flp-11* (see S3 Fig).

To determine which aspects of the egg-laying behavioral program may be subject to modulation by NLP-7 and FLP-11 peptides, we monitored the timing of egg-laying events in freely moving animals. In particular, we quantified the time intervals between consecutive active phases of egg-laying (intercluster intervals) as well as intervals between individual egg-laying events within an active phase (intracluster intervals). The duration of intercluster intervals in *nlp-7(lf);flp-11(lf)* double mutants is strikingly reduced compared with wild type or either single mutant (Fig 4A and 4B). In contrast, the duration of intracluster intervals is largely unaffected. Together, these results support a model where coordinated actions of NLP-7 and FLP-11 slow the rate of egg-laying by prolonging the length of the inactive phase (i.e. delaying transitions to the active phase).

### Local release of NLP-7 peptides from uv1 cells produces sustained inhibition of egg-laying

**Fig 5:**
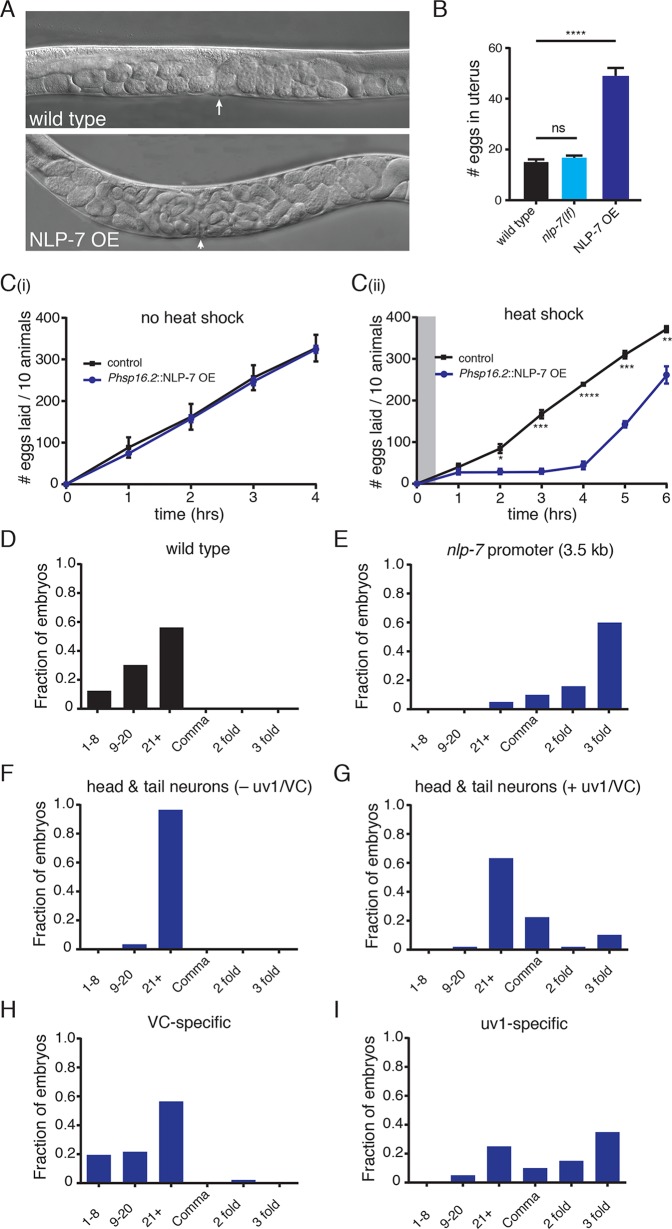
NLP-7 expression in the uv1 cells is required for the inhibition of egg-laying. (A) Representative DIC images of wild type (upper) and transgenic animals (*ufIs118)* stably expressing high levels of *nlp-7* genomic sequence (NLP-7 OE, lower). Arrows indicate position of the vulva. (B) Quantification of eggs *in utero*. Bars represent the mean ± SEM for each genotype. n≥20 for all measurements. ****p<0.0001, ANOVA with Sidak’s post hoc test. (C) Cumulative number of eggs laid over time in transgenic animals expressing the *nlp-7* genomic sequence under control of a heat shock promoter (*Phsp16*.*2*::NLP-7 OE) or control animals (non-transgenic siblings). Animals were either (i) maintained continuously at 20°C or (ii) exposed to heat shock (33°C, 30 mins). *p<0.05, **p<0.01, ***p<0.001, ****p<0.0001, t-test with Holm-Sidak’s correction for multiple comparisons. Gray shading indicates the timing and duration of heat shock. (D-I) Distribution of the developmental stages of eggs laid by either wild type adult hermaphrodites (D), or animals overexpressing *nlp-7* from a 3.5 kb *nlp-7* promoter (E), a 2.5 kb *nlp-7* promoter in which uv1 and VC fluorescence is not observed (F), a 2.5 kb *nlp-7* promoter in which uv1 and VC fluorescence is observed (G), a VC neuron specific promoter (*Plin-11*::*pes-10*) (H), or a uv1 promoter (*ocr-2*) (I). Animals overexpressing NLP-7 from the 3.5 kb promoter (E) lay a significantly higher fraction of late-stage embryos compared with wild type (p<0.0001, Fisher’s exact test). Exclusive expression in head and tail neurons produces no significant change (F), while there is a significant shift to later developmental stages when fluorescence is observed near the vulva (G) (p<0.0001, Fisher’s exact test). uv1-specific expression also produces a significant shift (p<0.0001, Fisher’s exact test) while VC-specific expression produces no significant difference. For each genotype, eggs laid within a 30 minute time period were evaluated. See also S3 Fig.

Our findings in the above rescue experiments prompted us to investigate whether increasing NLP-7 peptide levels may be sufficient to modify egg-laying behavior and, over time, lead to an egg-laying defective phenotype. To explore this possibility further, we expressed the *nlp-7* genomic sequence (including ~3.5 kb promoter region and 3’ UTR) at high levels in otherwise wild type animals. We found that egg-laying behavior is severely disrupted in animals stably expressing the *nlp-7* transgene (NLP-7 OE; *ufIs118*) (Fig 5A and 5B). In particular, these animals retain significantly more eggs *in utero* than the wild type (Fig 5B), and lay a significantly higher percentage of eggs in later stages of development (~95% in comma to 3-fold stage compared with 0 in the wild type)(Fig 5D and 5E). In contrast, overexpression of *flp-11* does not cause significant retention of eggs, but does rescue constitutive egg-laying in *nlp-7;flp-11* double mutants as noted above (Fig 3). The retention of eggs *in utero* with *nlp-7* overexpression provides evidence that chronic increases in NLP-7 peptide levels are sufficient to suppress egg-laying.

In order to investigate the time course over which NLP-7 may act in the control of egg-laying we next asked whether acute *nlp-7* expression is also sufficient to alter egg-laying. To address this question, we expressed *nlp-7* under the control of a heat-inducible promoter. At a constant temperature of 20°C, animals carrying this transgene do not show any obvious egg-laying defects (Fig 5C(i)) compared to non-transgenic controls. In contrast, exposing transgenic animals to a brief heat shock stimulus (33°C for 30 mins) produces a striking decrease in the rate of egg-laying. This decrease is evident within 2 hours following heat shock and lasted for a period of approximately four hours (Fig 5C(ii)). Further, these decreases are reversible, with animals increasing their egg-laying rates approximately 4 hrs following heat shock, such that total egg-laying approaches control levels by 6 hrs after heat shock (Fig 5C(ii)). These findings demonstrate that an acute rise in NLP-7 peptide levels is sufficient to block egg-laying in adult animals, and the time course of these effects suggests that the actions of NLP-7 are transient, consistent with the predicted modulatory effects of a neuropeptide.

We next pursued cell type-specific overexpression of *nlp-7* to decipher where peptide expression may be required for modulation of egg-laying. Our prior expression studies revealed that *nlp-7* is expressed in cells proximal to the vulva (uv1 and VC4/5) as well as in neurons near the head and tail that are distal to the core egg-laying circuit. An analysis of the *nlp-7* promoter region revealed a 2.5 kb fragment that drove predominant expression in the head and tail neurons, but provided more limited expression in the egg-laying circuit (fluorescence in egg-laying cells was visible in roughly 60% of animals). Using this 2.5 kb promoter to drive expression, we investigated egg-laying in the group of animals where we were able to confirm *nlp-7* expression was restricted to head and tail neurons. Selective expression in head and tail neurons does not produce an appreciable difference in the developmental stage of embryos at the time of egg-laying (Fig 5F), indicating that expression in these neurons alone is not sufficient for modulation of egg-laying activity. We also analyzed egg-laying in the group of animals for which we observed *nlp-7* expression in both head and tail neurons and the egg-laying cells (uv1 as well as more variable expression in the VC4 and VC5 neurons) using the shorter 2.5 kb *nlp-7* promoter fragment. We noted a significant increase in the fraction of eggs laid at later developmental stages in these animals (Fig 5G). To decipher which cells may be most critical, we used cell-specific promoters to drive overexpression of *nlp-7*. Surprisingly, despite the spatial proximity of the VC4 and VC5 neurons to the HSNs and vulval muscles, specific expression of *nlp-7* in the VC neurons (*lin-11* promoter fragment) does not alter egg-laying (Fig 5H). In contrast, specific expression in the uv1 cells (using *ocr-2* promoter and 3’ UTR) produces a striking shift in the developmental stage of eggs at the time of egg-laying (Fig 5I), similar to that observed using the 3.5 kb native *nlp-7* promoter. Together, our results support the idea that local NLP-7 release from uv1 cells is required for modulation of egg-laying activity. Further, the inability to elicit changes in egg-laying activity with expression in neighboring VC neurons may point toward a specialized role for the uv1 cells within the circuit.

### NLP-7 and FLP-11 reduce the activity of the egg-laying circuit

**Fig 6:**
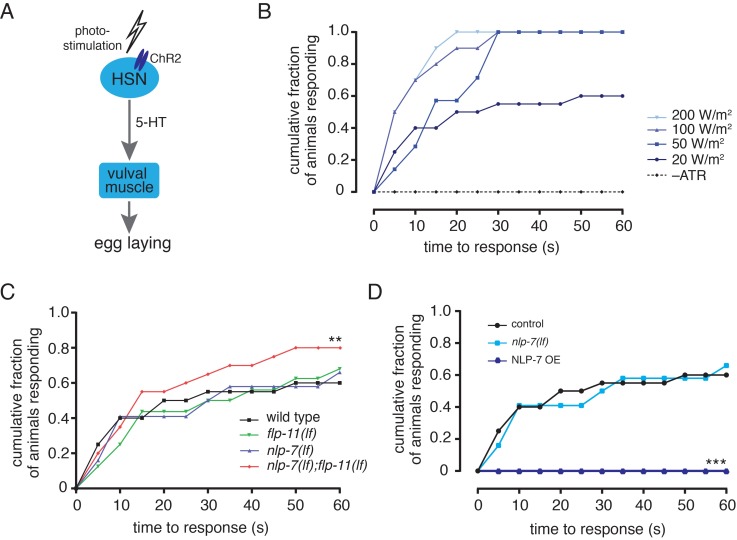
NLP-7 and FLP-11 regulate the activity of the egg-laying circuit. (A) Schematic diagram depicting optogenetic stimulation of the HSN neurons. Photostimulation of channelrhodopsin expressed in the HSNs (*Pegl-6a*::ChR2::YFP) elicits bouts of egg-laying [38](S3 Movie). (B) Cumulative fraction of animals that respond to blue light exposure over time. Latency to initial egg-laying event following light stimulus for varying light intensities. n = 10 for each condition.–ATR refers to control stimulation (50 W/m^2^) of the same genotype in the absence of retinal. (C) Cumulative plot of latency to initial egg-laying event following light stimulus (20 W/m^2^). Wild type, n = 20; *flp-11(lf)*,n = 16; *nlp-7(lf)*, n = 14; *nlp-7(lf);flp-11(lf)*, n = 20. **p<0.01, Wilcoxon matched-pairs test. (D) Cumulative plot of latency to initial egg-laying event following light stimulus (20 W/m^2^). ***p<0.001, Wilcoxon matched-pairs test.

Synaptic release from the HSN neurons onto vulval muscles is a key factor in the control of egg-laying activity. Therefore, to begin to address the question of how the behavioral effects of peptide modulation may be encoded within the egg-laying circuit, we next investigated whether neuropeptide signaling altered responsiveness in this core egg-laying pathway. Prior work showed that photostimulation of transgenic animals that express ChR2 in the HSNs is sufficient to elicit an active period of egg-laying [38] (Fig 6A). We therefore asked whether neuropeptide signaling affected bouts of egg-laying elicited by HSN photostimulation. We quantified egg-laying events in response to a 60 s period of light stimulation, measuring the time interval between the onset of light exposure and the initial egg-laying event as described previously [38](S3 Movie). As expected, control animals expressing ChR2 respond in a dose-dependent manner to blue light stimulation–the time interval to the initial egg-laying event decreased with increasing light intensities (Fig 6B). Using a light intensity that produced an intermediate response in controls (20 W/m^2^), we found that *nlp-7(lf);flp-11(lf)* double mutants are hypersensitive to light stimulation–there is a significant increase in the fraction of animals that respond to light stimulation over time compared with control animals or either single mutant (Fig 6C). In contrast, *nlp-7* overexpression completely blocks egg-laying responses to HSN photostimulation. Whereas almost 60% of wild type animals respond within 20 secs of blue light exposure, NLP-7 OE animals show no response within the 60 s period of light exposure (Fig 6D). Likewise, higher light intensities (up to 200 W/m^2^) do not elicit egg-laying responses in NLP-7 OE animals (none of the animals tested respond within 60 s, n = 30). Thus, altering peptide levels can have profound effects in this core HSN-muscle pathway, suggesting that NLP-7 and FLP-11 peptides likely inhibit egg-laying behavior either by acting to inhibit neurotransmitter release from the HSN neurons or by reducing responsiveness of egg-laying muscles.

### Elevating serotonin release or supplying exogenous serotonin reverses egg-laying defects caused by increased NLP-7

**Fig 7:**
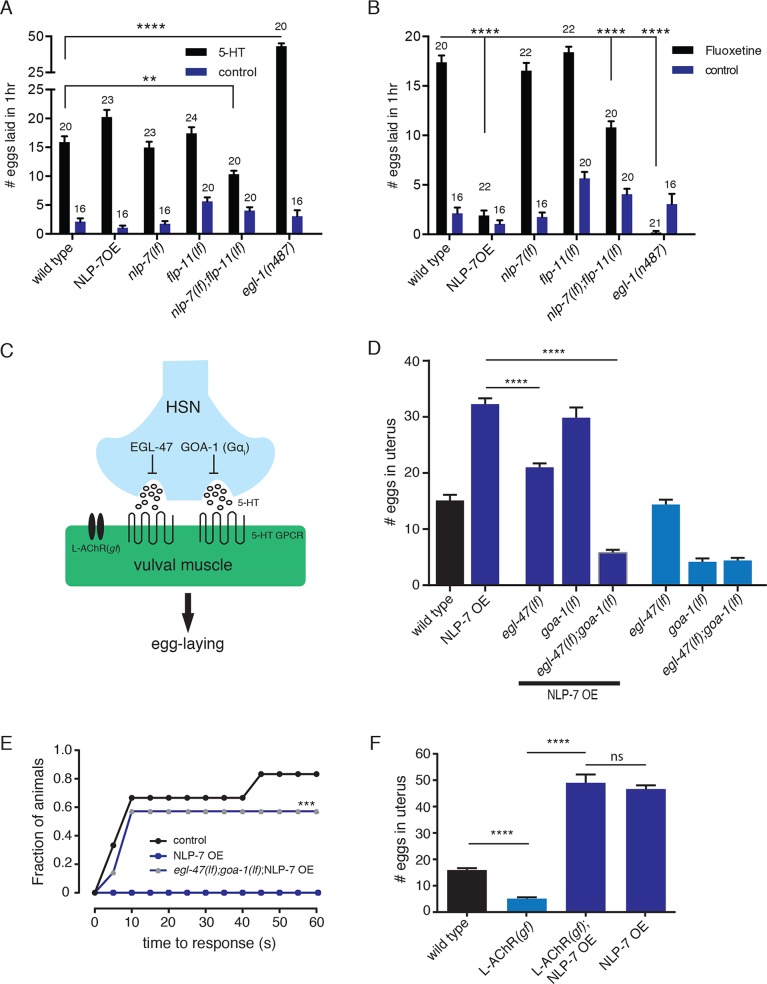
NLP-7 reduces the activity of the HSNs. (A) Quantification of eggs laid in 1 hour for the genotypes indicated, in either control buffer or in the presence of exogenous serotonin (7.5 mg/ml). Bars represent mean ± SEM for each condition. Numbers above bars indicate n for each condition. ****p<0.0001 ANOVA with Sidak’s post hoc test. For A and B, *egl-1* mutants in which the HSNs fail to develop are included as a control. 5-HT = serotonin. See also S4 Fig for comparison with egg-laying on plates. (B) Quantification of eggs laid in 1 hour for the genotypes indicated in either control buffer or in the presence of the serotonin reuptake inhibitor fluoxetine (0.5 mg/ml). Bars represent mean ± SEM for each condition. Numbers above bars indicate n for each condition. **p<0.01, ****p<0.0001 ANOVA with Sidak’s post hoc test. (C) Schematic of genetic manipulations used to modify egg-laying activity. See text for details. (D) Quantification of eggs *in utero*. Bars represent the mean ± SEM for each genotype. n≥20 for all measurements. ****p<0.0001, ANOVA with Sidak’s post hoc test. (E) Cumulative plot of latency to initial egg-laying event following light stimulus (50 W/m^2^) the genotypes indicated expressing *Pegl-6a*::ChR2::YFP. n≥15 for all measurements. ***p<0.001, Wilcoxon matched pair test. (F) Quantification of eggs retained *in utero*. Bars represent mean ± SEM for each genotype. L-AChR*(gf)* refers to stable muscle-specific expression of AChRs with elevated ACh responsiveness [45]. n≥15, ****p<0.0001, ANOVA with Sidak’s post hoc test.

Based on our above optogenetic analysis, we used multiple approaches to explore mechanisms by which NLP-7 and FLP-11 peptides regulate HSN-muscle signaling and egg-laying. The HSNs are primarily serotonergic and we therefore first tested whether pharmacological approaches that alter serotonin levels would normalize egg-laying in NLP-7 OE animals. Prior work demonstrated that the egg-laying defects produced by serotonin deficiency are reversed by supplying exogenous serotonin [24,39]. We found that serotonin exposure stimulates egg-laying similarly in both wild type animals and neuropeptide-deficient single mutants. Serotonin exposure elicits fewer egg-laying events in *nlp-7;flp-11* double mutants, likely due to the fact that these animals display constitutive egg-laying and retain fewer eggs *in utero* (Fig 3D). Strikingly, serotonin exposure also promotes egg-laying in NLP-7 OE animals, suggesting that egg-laying defects in these animals might arise through a deficiency in serotonin levels (Fig 7A). Consistent with this interpretation, we found that the serotonin reuptake inhibitor fluoxetine elicits far less egg-laying activity in NLP-7 OE animals compared with wild type (Fig 7B). Fluoxetine exposure also elicits a reduced number of egg-laying events in *nlp-7;flp-11* double mutants compared with wild type, again likely because these animals retain fewer eggs *in utero*.

We next pursued several genetic approaches to ask whether HSN activity and serotonin release are regulated by neuropeptide signaling. We used available loss-of-function mutations in *egl-47* and *goa-1*, two genes that act primarily in the HSNs to inhibit egg-laying, to elevate HSN signaling [23,40–42] (Fig 7C). *egl-47* encodes a seven transmembrane protein bearing homology to *Drosophila* gustatory receptors [43] while *goa-1* encodes Gα_o_, the only inhibitory G-protein subunit expressed in the HSNs. Mutation of *egl-47* partially reverses NLP-7 inhibition of egg-laying (Fig 7D). Though loss of *goa-1* produces constitutive egg-laying in otherwise wild type animals, we did not observe appreciable differences in egg-laying between control NLP-7 OE animals and NLP-7 OE animals carrying a *goa-1(lf)* mutation. Combined mutation of *egl-47* and *goa-1* however completely reverses the effects of *nlp-7* overexpression, eliciting a constitutive egg-laying phenotype comparable to that of *goa-1(lf)* single mutants (Fig 7D). We next examined whether combined mutation of *egl-47* and *goa-1* in NLP-7OE animals restored responsiveness to HSN photostimulation. Egg-laying responses elicited by ChR2 activation of the HSNs approach wild type levels in these animals (Fig 7E), providing additional evidence that NLP-7 inhibition of egg-laying is reversible with increases in HSN signaling. Finally, we asked whether increasing muscle synaptic activation could suppress NLP-7 mediated inhibition of egg laying. Cholinergic transmission at synapses between VC neurons and egg-laying muscles operates in parallel with HSN signaling onto muscles to control their excitability. Vulval muscles express acetylcholine receptors that are responsive to the nematode-specific cholinergic agonist levamisole, and exposure to exogenous levamisole stimulates egg-laying [44]. We increased cholinergic synaptic activation of muscles by expressing a gain-of-function muscle acetylcholine receptor (L-AChR*(gf)*) [45]. Transgenic expression of L-AChR*(gf)* in otherwise wild type animals increases egg-laying activity, producing a constitutive phenotype (Fig 7F). In contrast, expression of L-AChR*(gf)* in NLP-7 OE animals does not appreciably change the rate of egg-laying, indicating that elevated cholinergic synaptic activation of muscles cannot reverse the inhibition produced by increased levels of NLP-7 signaling. Interestingly, NLP-7 OE animals expressing the L-AChR*(gf)* transgene show enhanced egg-laying with a supply of exogenous serotonin (–5-HT: 0.4±0.3 eggs/hour; +5-HT: 12.8±1.9 eggs/hour, p<0.0001, t-test), consistent with the notion that the production or release of serotonin may be impaired in NLP-7 OE animals.

As noted above, FLP-11 expression is sufficient for rescue of constitutive egg-laying in *nlp-7;flp-11* double mutants, but overexpression does not elicit sustained inhibitory effects. Therefore, we were unable to pursue a similar genetic analysis of FLP-11 signaling. This could be due to a functional property of the FLP-11 signaling pathway or may indicate that our strategies for increasing neuropeptide expression were less effective for FLP-11. Nonetheless, our findings for NLP-7 argue that these peptides act primarily to control levels of serotonin secretion from the HSNs. Prior work showed that HSNs exhibit spontaneous Ca^2+^ oscillations even in the absence of synaptic inputs [46]. Therefore, our findings suggest a model where local neuropeptide release from the uv1 cells may control circuit activity, in part by shaping the timing of vesicular serotonin release from the HSNs.

### NLP-7 regulates synaptic vesicle abundance in the HSN neurons

To gain additional support for peptide regulation of HSN transmission, we next examined the distribution of synaptic vesicles at HSN synapses onto vulval muscles. Numerous previous studies have employed the synaptic vesicle marker synaptobrevin (SNB-1) to investigate alterations in the distribution of synaptic vesicles in the HSNs [47–51]. Therefore for our analysis, we used a transgenic strain in which GFP-tagged synaptobrevin (SNB-1::GFP) is stably coexpressed specifically in the HSN neurons with the fluorescent reporter DsRed2 [50].

**Fig 8:**
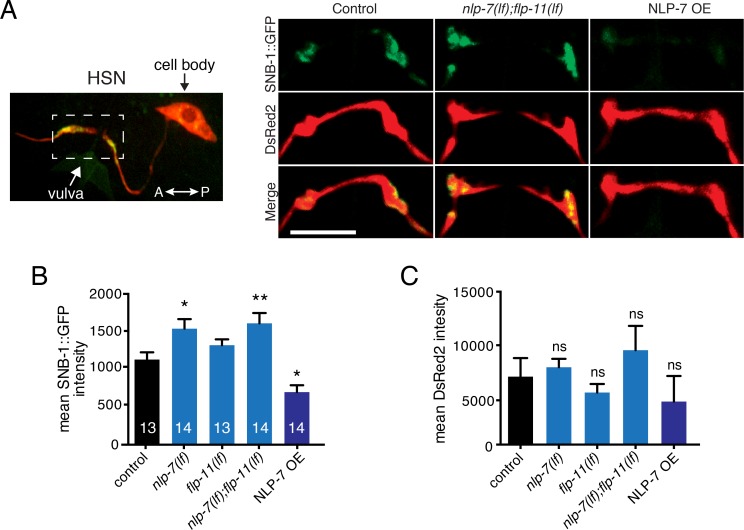
NLP-7 signaling regulates SNB-1::GFP abundance at HSN synapses. (A) Left, confocal image of HSN cell body and synaptic regions. Dashed white box indicates approximate area of synaptic regions detailed at right and used for analysis. Right, representative confocal images of HSN presynaptic varicosities in transgenic animals expressing the synaptic vesicle marker SNB-1::GFP and DsRed2 in the HSNs (*vsIs103*, *Ptph-1*::SNB-1::GFP; *Ptph-1*::DsRed2) [50] for the genotypes indicated. (B, C) Quantification of average SNB-1::GFP (B) or DsRed2 (C) intensity in synaptic region of the HSNs for the genotypes indicated. Bars represent mean ± SEM for each condition. See also S5 and S6 Figs for additional quantification of SNB-1::GFP and the active zone marker GFP::SYD-2. SNB-1::GFP fluorescent intensity is significantly increased or decreased in HSN synaptic regions of *nlp-7* mutants or NLP-7 OE animals respectively (see model in S7 Fig). Numbers in bars indicate the n for each condition. *p<0.05, **p<0.01, ANOVA with Sidak’s post hoc test. Scale bar, 7 μm.

In wild type animals, the HSN process forms two to four synaptic varicosities within 10μm of the vulval opening where synaptic contacts with the Vm2 vulval muscles are located [51]. We did not observe significant changes in the morphological features of the HSN neurons in either of the neuropeptide single mutants or in *nlp-7;flp-11* double mutants. Likewise, the total synaptic volume (S5A Fig) and the number of synaptic varicosities (S5B Fig) within the HSN process are unaffected by single or combined deletion of the *flp-11* and *nlp-7* neuropeptide precursors, suggesting that the sizes of the synaptic region are not altered. However, we noted a significant increase in the intensity of SNB-1::GFP fluorescence in both *nlp-7(lf)* single mutants and *nlp-7(lf);flp-11(lf)* double mutants (Fig 8B), suggesting that synaptic vesicle abundance in the HSN neurons is increased in these animals. In principle, the increase in vesicle abundance could affect either egg-laying activity during the active phase or the length of the quiescent period. Despite increased SNB-1::GFP, we did not observe a significant change in the number of egg-laying events/active phase for either *nlp-7(lf)* single mutants and *nlp-7(lf);flp-11(lf)* double mutants (wild type: 3.8±0.3; *nlp-7(lf)*: 4.1±0.3; *nlp-7(lf);flp-11(lf)*: 3.2±0.2), supporting the idea these peptides act primarily to increase the length of the quiescent period. Deletion of *flp-11* did not produce an appreciable difference in SNB-1::GFP intensity, suggesting that HSN regulation may be primarily mediated through NLP-7 peptides, while FLP-11 peptides may modulate egg-laying activity via another mechanism. Conversely, we noted a significant decrease in SNB-1::GFP fluorescent intensity in NLP-7 OE animals (Fig 8A and 8B), though the total volume of the synaptic region remained unchanged (S5A Fig). Finally, DsRed2 fluorescent intensity (expressed under the same promoter as SNB-1::GFP) in either the synaptic region or the HSN cell body is not significantly affected by combined peptide deletion or by NLP-7 overexpression (Fig 8C and S5E Fig), providing evidence that the changes in SNB-1::GFP fluorescence we observe do not arise via transcriptional regulation of the reporter. Similarly, neither localization or expression of the active zone marker GFP::SYD-2 were appreciably altered by peptide deletion or overexpression [52] (S6 Fig), providing additional evidence that synapse organization is not affected by altering neuropeptide signaling. In contrast, SNB-1::GFP fluorescent intensity in the HSN cell bodies is significantly reduced by NLP-7 overexpression, similar to the synaptic region (S5C and S5D Fig). Together, our analysis supports a model where neuropeptide secretion from the uv1 cells modulates egg-laying activity, at least in part by limiting the abundance of serotonergic synaptic vesicles at HSN synapses. As reduced synaptic vesicle trafficking to synapses might be expected to lead to an accumulation of synaptic vesicles in the cell body, we propose that this regulation occurs via an alternate mechanism, perhaps through direct or indirect regulation of synaptic vesicle biogenesis.

## Discussion

Neuromodulatory signaling enables hardwired circuits to maintain flexibility for generating alternate activity patterns and behaviors in response to either changes in environmental conditions or internal state of the organism. Gaining a mechanistic understanding of these processes *in vivo* however has remained a challenge. Our studies here demonstrate that neuropeptide modulation of neurotransmitter release controls the temporal pattern of *C*. *elegans* egg-laying activity. While prior work has provided examples of neuromodulatory control of egg-laying in response to external environmental factors [10,46,53,54], our work demonstrates that neuromodulatory signaling is central for establishing the timing of egg-laying activity even under relatively constant favorable external conditions.

Prior work suggested that the neurosecretory uv1 cells may terminate egg-laying, perhaps by sensing the passage of eggs through the vulva [18,29]. Our work supports this model and extends it in several important aspects. First, we show that activating or inhibiting the uv1 cells produces predictable changes in the periodicity of egg-laying. Second, we identify two neuropeptide precursors, NLP-7 and FLP-11, that are expressed in uv1 cells and work cooperatively to set the duration of inactive phases during egg-laying. For both neuropeptide precursors, specific expression in the uv1 cells is sufficient to slow the rate of egg-laying, suggesting that local secretion solely from uv1 cells can account for their effects *in vivo*. Third, we show that *nlp-7* overexpression produces robust and sustained inhibition of egg-laying, and provide evidence that this occurs through negative regulation of serotonin signaling from the HSN neurons. Finally, genetic ablation or overexpression of *nlp-7* produce opposing effects on synaptic vesicle abundance in the HSN neurons. Genetic alteration of *flp-11* levels does not reproduce these effects, suggesting *flp-11* may act via another mechanism or target another cell type in the circuit. Together, our findings support a model where peptides derived from NLP-7 and FLP-11 are coordinately secreted from uv1 cells to modify egg-laying circuit activity and shape the temporal organization of egg-laying behavior. We propose that the secretion of NLP-7 and FLP-11 peptides from uv1 cells promote egg-laying quiescent periods by reducing serotonin release from the HSN neurons. This is achieved, at least in part, by regulating the abundance of serotonergic synaptic vesicles in the HSN neurons (S7 Fig). Prior work showed that neuropeptides encoded by the *flp-1* gene promote the onset of egg-laying active phases [10]. It is therefore appealing to speculate that the NLP-7/FLP-11 and FLP-1 neurotransmitter systems act antagonistically to shape egg-laying behavior. One intriguing possibility is that FLP-1 peptides primarily act in response to sensory stimuli (such as food availability) to alter an intrinsic rhythm established through the actions of NLP-7/FLP-11 signaling.

Our studies indicate that local neuropeptide signaling plays a central role in establishing behavioral rhythmicity during egg-laying. In particular, we find that cell-specific expression of *flp-11* or *nlp-7* is sufficient to rescue egg-laying defects or, in the case of *nlp-7*, produce sustained inhibition of egg-laying. Interestingly, cell-specific expression of *nlp-7* in VC motor neurons did not reproduce these effects, even though these neurons are located in close proximity to both HSN neurons and egg-laying muscles. This finding points toward the idea that the uv1 cells perform specialized neurosecretory functions in halting egg-laying. For example, uv1 cells may be specialized for properly coordinating the timing of neuropeptide release with egg-laying activity or perhaps possess expanded capacity for neuropeptide release compared with neighboring neurons. Unfortunately, it has proven difficult to assess uv1 function directly. These cells are an essential structural component of the egg-laying anatomy, limiting the value of ablation studies or mutants deficient in uv1 development for addressing questions about function [29,55]. Prior studies exploited a dominant mutation in the TRPV channel *ocr-2* to strongly implicate uv1 cells in the regulation of egg-laying, but mechanistic interpretation of these studies is complicated by our limited understanding of the effects of the mutation on channel functional properties [29]. We used expression of a histamine gated chloride channel to inhibit uv1 activity. Prolonged inhibition of the uv1 cells in adult animals significantly reduces the length of the inactive phase of egg-laying. A recent study found that calcium transients occur in uv1 cells immediately following egg-laying events during an active phase [18], suggesting that repetitive stimulation of uv1 cells may be required for inhibition of egg-laying. We speculate that each egg-laying event may trigger release from the uv1 cells, ultimately leading to local increases in peptide levels sufficient to terminate the active phase and delay re-entry into the subsequent active phase. This model is supported by several observations: (1) ChR2 depolarization of uv1 cells delays subsequent egg-laying events in an active phase and even terminates the active phase in a subset of animals. (2) The effects of uv1 stimulation are almost completely eliminated in double mutants lacking NLP-7 and FLP-11 peptides. (3) uv1-specific rescue or overexpression of *flp-11* and *nlp-7* demonstrate that high levels of either peptide are sufficient to inhibit egg-laying. Moreover, induced expression of NLP-7 peptides in adults slows egg-laying within two hours and these effects begin to reverse by four hours. uv1 cells may share interesting parallels with oxytocin (OT) neurosecretory cells of the hypothalamus that are involved in the control of lactation. Secretion of OT (and subsequently milk ejection) is not temporally locked to the initiation of suckling but instead must surpass a certain threshold during suckling for a burst of OT release and milk ejection to occur [56,57]. Similarly, we speculate that uv1 cells require a buildup of egg-laying events during an active phase to achieve sufficient levels of NLP-7 and FLP-11 peptides to terminate an active phase. These neuropeptides may also work in concert with other modes of uv1 signaling. For example, genetic disruption of tyraminergic signaling produces defects in egg-laying and prior work showed that tyramine was present in the uv1 cells [26]. As is the case for *nlp-7;flp-11* double mutants, the effects of tyramine deficiency are incomplete, suggesting that these signals may cooperate to alter circuit performance in complex ways.

While our work supports the idea that neuropeptide secretion from uv1 cells is key for proper temporal organization of egg-laying, the nature of the sensor that triggers peptide secretion remains an open question. All of our studies are performed under constant favorable external conditions. Therefore, peptide secretion from uv1 cells is likely triggered by sensing a change in the internal state of the organism, perhaps by sensing egg-laying status. Based on their anatomical position and expression of mechanosensory TRPV channels, the uv1 cells have been proposed to sense the mechanical load of the uterus [29]. Consistent with this idea, recent evidence indicates that uv1 cells are mechanically deformed with the passage of eggs through the vulva [18]. Thus, mechanical deformation of the uv1 cells may directly trigger rises in intracellular Ca^2+^ and peptide secretion.

Our genetic analysis of *nlp-7* overexpression provides evidence that NLP-7 peptides act primarily at the level of the HSNs to inhibit egg-laying. We found that combined mutation of *egl-47* and *goa-1* completely suppress the effects of *nlp-7* overexpression. Prior work demonstrated that each of these genes mediate their effects on egg-laying by acting primarily in the HSN neurons [41], although *goa-1* expression in vulval muscles or uv1 cells may also contribute [29,40]. Nonetheless, it is difficult to conclusively demonstrate that the HSN neurons are the sole site of action for these peptides in the egg-laying circuit because high-affinity receptors for NLP-7 peptides have not yet been identified, and the cellular expression of several putative FLP-11 receptors remains uncharacterized. *egl-6* encodes the sole GPCR that has been previously demonstrated to show expression in the HSN neurons, but NLP-7 peptides do not activate EGL-6 *in vitro* [54]. Though a precise mechanism for EGL-47 action remains unclear, our finding that mutation of *egl-47* partially restores egg-laying in NLP-7 OE animals raises the intriguing possibility that EGL-47 may act directly as a receptor for NLP-7 peptides. *egl-47* encodes a seven-transmembrane protein related to *Drosophila* gustatory receptors [43,58]. Initial studies suggested that *egl-47* may be an orphan GPCR [41], while more recent studies suggest that EGL-47 inhibition of egg-laying may occur through activation of an associated chloride channel [51]. While gain-of-function *egl-47* mutation inhibits egg-laying, our studies here are the first to demonstrate a phenotype associated with *egl-47* loss of function. In addition, our findings reveal that the egg-laying defects of NLP-7 overexpressing animals are completely suppressed only by combined mutation of *egl-47* and *goa-1*. This observation points towards the interesting possibility that multiple receptors might act downstream of NLP-7 signaling–perhaps EGL-47 in combination with GOA-1 (G_i/o_)-coupled GPCR(s). Further characterization of EGL-47 and other potential NLP-7 receptors is an important subject for future studies. Several potential FLP-11 receptors have been identified to date [37,59–61], but expression of these receptors in the egg-laying circuit has not yet been demonstrated. In our studies, FLP-11 expression in the uv1 cells was sufficient for rescue of constitutive egg-laying in *nlp-7;flp-11* double mutants, but overexpression did not produce obvious defects, preventing pursuit of a genetic suppressor approach to identify the GPCR(s) involved.

Our studies also address the question of how peptides secreted from the uv1 cells modulate egg-laying. Our analysis of SNB-1::GFP fluorescence in the HSN neurons offers support for the idea that NLP-7 peptides act, at least partially, by modulating HSN transmission onto egg-laying muscles. We observed an increase in SNB-1::GFP fluorescence at HSN synapses in *nlp-7(lf);flp-11(lf)* mutants, and a strong decrease with *nlp-7* overexpression. Because the total synaptic volume and number of varicosities are unaffected, we propose that these fluorescence changes reflect altered synaptic vesicle density. We therefore interpret our findings to indicate that peptidergic signaling alters synaptic vesicle abundance in the HSN neurons. This interpretation is supported by our HSN photostimulation studies where we found that *nlp-7(lf);flp-11(lf)* double mutants exhibit enhanced egg-laying responsiveness to HSN depolarization, while NLP-7 OE animals are resistant. Prior work has identified mechanisms for intrinsic regulation of HSN activity [38,46,51,62]. In particular, the potassium channel IRK-1 is important for inhibition of HSN activity by the EGL-6 GPCR. Although acting independently of EGL-6, NLP-7 and FLP-11 peptides might similarly exert their effects by inhibiting HSN activity. Our analysis of SNB-1::GFP fluorescence is not however consistent with the peptides acting solely via this mechanism. For example, if NLP-7 peptides inhibited egg-laying simply by decreasing HSN activity, we might expect an increase in SNB-1::GFP fluorescence at synapses due to a reduced rate of vesicular release. Surprisingly, we observe that NLP-7 mediated inhibition of egg-laying is associated with a strong decrease in SNB-1::GFP intensity, suggesting other processes contribute. One possibility is that chronic peptidergic inhibition of the HSNs leads to a compensatory decrease in the production of synaptic vesicles. Alternatively, neuropeptide signaling may directly modulate the rate of synaptic vesicle trafficking to HSN synapses. This possibility appears unlikely because we do not observe increased SNB-1::GFP intensity in the HSN cell bodies of NLP-7 OE animals, as would be expected if vesicles were accumulating in the HSN soma. Finally, peptidergic signaling may directly alter rates of HSN synaptic vesicle recycling or biogenesis. A previous report demonstrated that serotonin expression in the HSNs is altered by G-protein signaling [50]. Our findings may suggest the presence of a parallel mechanism in the HSNs for regulating vesicle production. This would be consistent with our observation that SNB-1::GFP intensity is decreased at both HSN synapses and in HSN cell bodies of NLP-7 OE animals. Interestingly, we also observed that SNB-1::GFP fluorescent intensity is significantly increased at HSN synapses in *nlp-7* single mutants, but these animals do not display significant egg-laying defects and likewise are not hypersensitive to HSN photostimulation. These results argue that changes in HSN synaptic vesicle abundance alone cannot fully account for the actions of FLP-11 and NLP-7 peptides in the egg-laying circuit.

In summary, our results demonstrate a mechanism by which local neuropeptide secretion from a group of non-neuronal cells produces relatively long-lived inhibition of circuit activity and behavioral quiescence by modulating serotonergic transmission. HSN motor neurons exhibit spontaneous activity, and serotonin release from the HSNs is associated with a transition from the inactive to active phase of egg-laying [9,46]. We propose that cycles of neuropeptide release from the uv1 cells reversibly inhibit HSN transmission and structure the timing of egg-laying by both promoting inactive phases and preventing rapid re-entry into active phases. More broadly, our studies elucidate a mechanism by which neuromodulatory systems modify circuit activity to specify the timing of transitions between opposing behavioral states. Advancing our knowledge of how neuropeptides and other modulators act in the context of the circuits in which they are endogenously released will be critical in ongoing efforts to understand how alternate behavioral states, for example those underlying mood or arousal, are encoded.

## Materials and methods

### Strains

All nematode strains were maintained at 20°C on agar nematode growth media plates seeded with *E*. *Coli* OP50. The wild type reference animals for all cases are the N2 Bristol strain. The following strains were used or generated in this work: IZ2539: *ufIs160*[*Pocr-2*::*HisCl*::*SL2*::*GFP*:*ocr-2 3’ UTR*, *Plgc-11*::*mCherry*], LX2047: *lite-1(ce314);lin-15(n765ts);vsIs189*[*Pocr-2*::*ChR*::*YFP*::*ocr-2* 3’UTR], IZ2634: *nlp-7(tm2984);flp-11(tm2706);lite-1(ce314);lin-15(n765ts);vsIs189*, NY2040: *ynIs40*[p*flp-11*::GFP], IZ1130: *lin-15(n765ts);ufEx378* [p*nlp-7*::*nlp-7*::*SL2*::*mCherry*], IZ1135: *nlp-7(tm2984)*, IZ2022: *flp-11(tm2706)*, IZ1589: *nlp-7(tm2984);flp-11(tm2706)*; IZ2263: *nlp-7(tm2984);flp-11(tm2706);ufEx792*[*Pnlp-7*::*nlp-7*::*nlp-7* 3’UTR, *Plgc-11*::GFP], IZ2407: *nlp-7(tm2984);flp-11(tm2706);ufEx876*[*Pflp-11*::*flp-11*::*flp-11* 3’ UTR, *Plgc-11*::GFP], IZ2834: *nlp-7(tm2984);flp-11(tm2706);ufEx1099 [Pocr-2*::*nlp-7*::*ocr-2 3’ UTR*, *Pocr-2*::*mCherry*::*ocr-2 3’UTR*, *Plgc-11*::*GFP]*, IZ1823: *nlp-7(tm2984);flp-11(tm2706);ufEx582*[*Pocr-2*::*flp-11*::*ocr-2 3’UTR*, *Pocr-2*::*mCherry*::*ocr-2 3’UTR*, *Plgc-11*::*GFP*], LX1836: *lite-1(ce314);lin-15(n765ts);wzIs30*[p*egl-6*::ChR::YFP], IZ1587: *nlp-7(tm2984); lite-1(ce314);lin-15(n765ts);wzIs30*, IZ2008: *flp-11(tm2706); lite-1(ce314);lin-15(n765ts);wzIs30*, IZ2007: *nlp-7(tm2984);flp-11(tm2706); lite-1(ce314);lin-15(n765ts);wzIs30*, IZ1614: *ufIs118;lite-1(ce314);lin-15(n765ts);wzIs30*, IZ1236: *ufIs118 [Pnlp-7*::*nlp-7*::*nlp-7 3’UTR*, *Plgc-11*::*GFP]*, IZ2109: *ufEx730*[*Phsp16*.*2*::*nlp-7*], IZ1594: *ufEx521*[*Pocr-2*::*nlp-7*::*ocr-2 3’UTR*, *Plgc-11*::*mCherry*], IZ2006: *ufEx681*[*Plin-11*::*pes-10*::*nlp-7*, *Plin-11*::*pes-10*::*mCherry*, *Plgc-11*::*GFP*], IZ1692: *ufEx548*[*Pnlp-7(2*.*5kb)*::*nlp-7*, *Pnlp-7(2*.*5kb)*::*mCherry*, *Plgc-11*::*GFP*], RB850: *egl-47(ok677)V*, IZ1235: *egl-47(ok677);ufIs118*, IZ1324: *goa-1(n1134);ufIs118*, IZ2096: *egl-47(ok677)*, *goa-1(n1134);ufIs118*, MT2426: *goa-1(n1134)*, IZ2683: *egl-47(ok677)*, *goa-1(n1134)*, IZ2474: *egl-47(ok677)*, *goa-1(n1134);ufIs118;lite-1(ce314);lin-15(n765ts);wzIs30*, IZ236: *ufIs6 [Pmyo-3*:: *unc-38(V/S)*, *Pmyo-3*::*unc-29(L/S)*, *Pmyo-3*:: *lev-1(L/S)]*, IZ1274: *ufIs6;ufIs118*, LX967: *lin-15(n765ts);vsIs103*, IZ1881: *nlp-7(tm2984); lin-15(n765ts);vsIs103*, IZ1880: *flp-11(tm2706); lin-15(n765ts);vsIs103*, IZ1884: *nlp-7(tm2984);flp-11(tm2706); lin-15(n765ts);vsIs103*, IZ1812: *ufIs118; lin-15(n765ts);vsIs103*, BL5752: *inIs181(Pida-1*::IDA-1::GFP*);inIs182(Pida-1*::IDA-1::GFP*)*, IZ2681: *inIs181;inIs182;ufEx378*, TV38: *wyIs12 [Punc-86*:: *GFP*::*SYD-2*, *Podr-1*::*GFP]*, IZ2818: *wyIs12;ufIs118*, IZ2819: *wyIs12;nlp-7(tm2984);flp-11(tm2706)*.

### Molecular biology and transgenes

*HisCl*: A fragment containing the coding sequence of HisCl SL2 trans-spliced with GFP was ligated into pKMC281 (a gift from the Koelle lab) between the *ocr-2* promoter and the *ocr-2* 3’ UTR to generate pNB41 (*pocr-2*::*HisCl*::*SL2*::*GFP*::*ocr-2 3’UTR*). This was injected (100 ng/μl) into N2 animals along with the coinjection marker pBB107 (*Plgc-11*::*mCherry*, 30 ng/μl). The resulting extrachromosomal array was stably inserted by X-ray integration to generate the transgene *ufIs160* and outcrossed four times to wild type.

*nlp-7 expression pattern*: pCL17 (*Pnlp-7*::*nlp-7*::*SL2*::*mCherry*, 50 ng/μl) was injected into *lin-15(n765ts)* animals along with the coinjection marker pL15EK (*lin-15(+)*, 50 ng/μl).

*nlp-7 and flp-11 rescue*: For *nlp-7* rescue, a 5104 bp PCR product containing the *nlp-7* promoter, genomic locus and the 3’UTR (-3448 bp to +1656 bp relative to the transcriptional start) was injected at 50 ng/μl into strain IZ1589 along with the coinjection marker pHP6 (*Plgc-11*::*GFP*, 30 ng/μl). For *flp-11* rescue, a 3240 bp PCR product containing about 2.5 kb of the *flp-11* promoter, genomic locus and the 3’UTR (-2518 bp to +722 bp relative to the transcriptional start) was injected (100 ng/μl) into strain IZ1589 along with pHP6 (30 ng/μl).

*NLP-7 overexpression*: The NLP-7 OE strain (*ufIs118*) was generated by microinjection and subsequent X-ray integration of a 5104 bp PCR product (injected at 100 ng/μl) containing the *nlp-7* promoter (about 3.5 kb), genomic locus and the 3’UTR (-3448 bp to +1656 bp relative to the transcriptional start) along with pHP6 (*Plgc-11*::*GFP*, 50 ng/μl) as coinjection marker. The integrated strain was outcrossed five times with wild type.

*Heat shock overexpression of nlp-7*: pNB35 (*Phsp16*.*2*::*nlp-7*, 100 ng/μl) was injected into N2 animals along with pHP6 (*Plgc-11*::*GFP*, 30 ng/μl) as coinjection marker.

### Cell specific overexpression of nlp-7

*uv1*: The *nlp-7* genomic sequence was ligated into pKMC281 (a gift from the Koelle lab) between the *ocr-2* promoter and the *ocr-2* 3’ UTR to generate pNB28 (*Pocr-2*::*nlp-7*::*ocr-2 3’UTR*). A PCR product amplified from this plasmid was injected at 100 ng/μl into N2 animals together with pBB107 (30 ng/μl).

*VC*: A fragment containing the *lin-11* enhancer region fused to the *pes-10* basal promoter was PCR amplified from pDM4 and ligated into pENTR/D-TOPO vector to generate a gateway entry vector. It was recombined with gateway destination vectors containing the *nlp-7* genomic sequence and mCherry coding sequence to generate pNB14 and pNB15. pNB14 (*Plin-11*::*pes-10*::*nlp-7*, 100 ng/μl) was coinjected with pNB15 (*Plin-11*::*pes-10*::*mCherry*, 80 ng/μl) into N2 animals together with pHP6 (30 ng/μl).

*Head and tail neurons*: A 2537 bp fragment containing the *nlp-7* promoter (-2537bp relative to transcriptional start) was amplified from genomic DNA and ligated into pENTR/D-Topo vector to generate a Gateway entry vector. This was recombined to Gateway destination vectors containing the *nlp-7* genomic sequence and mCherry coding sequence to generate pNB24 (*Pnlp-7(2*.*5kb)*::*nlp-7*) and pNB25 (*Pnlp-7(2*.*5kb)*::*mCherry*) respectively. pNB24 (100 ng/μl) and pNB25 (50 ng/μl) were coinjected into N2 animals together with pHP6 (30 ng/μl).

### Behavioral assays

#### Quantification of eggs in uterus

Age-matched adults were obtained by collecting late fourth larval stage (L4) animals and culturing at 20°C for 30 hrs (except in Fig 7 where synchronized adults 24 hrs after the L4 stage were used). For each strain analyzed, animals were individually dissolved in 25% sodium hypochlorite, and their eggs, which survived because of their protective eggshells, were quantified.

#### Embryo staging assay

To score the developmental stage of newly laid eggs, age-matched adults (30 hrs after the late L4 stage) were transferred to fresh nematode growth medium plates (15 animals per plate), allowed to lay eggs for 30 mins and removed. Eggs laid on the plates were examined by a high power dissecting microscope and categorized as described in Ringstad and Horvitz, 2008.

#### Analysis of temporal pattern of egg-laying

Single one-day old (24–30 hrs after the L4 stage) adult animals were placed individually on NGM agar plates and videotaped at room temperature for 4–5 hours at a rate 0.5 frames/s. Intervals between clusters of egg-laying (intercluster intervals) and intervals between individual egg-laying events within a cluster (intracluster intervals) were determined by video analysis and manually scoring the timing of egg-laying events.

#### HisCl experiments

Histamine plates were made by diluting histamine dihydrochloride (Sigma Aldrich) into NGM agar media cooled to 55°C, to a final concentration of 50 mM. Plates were stored at 4°C and used within 2 weeks. To quantify the number of eggs *in utero*, age matched adults (24 hrs after the L4 stage) were placed on seeded NGM plates with or without histamine. After 6 hrs at room temperature, animals were individually bleached and the number of eggs retained *in utero* were counted. To analyze the timing of egg-laying events, one-day old adults were placed on histamine plates. After 1 hr of histamine exposure, animals were videotaped for 3–5 hrs while on the same histamine plates. The timing of individual egg-laying events was determined by manual video analysis. Control animals were treated similarly except that they were placed on plates lacking histamine.

#### Pharmacological assays

Individual staged adult animals were placed in 50 μl of low salt M9 buffer, or M9 containing 7.5 mg/ml serotonin or 0.5 mg/ml fluoxetine. After 1 hour in each condition, the number of eggs laid by each animal was quantified.

#### Channelrhodopsin experiments

Synchronized young adult animals were transferred to retinal plates for roughly 18 hours prior to experiment. To prepare retinal plates, an overnight culture of OP50 was mixed with all-*trans* retinal to a final concentration of 100 μM and 150 μl of the mix was seeded onto individual NGM agar plates. Plates were stored at 4°C under dark conditions and used within one week. Photostimulation experiments were conducted using a fluorescent dissecting microscope (Zeiss steREO Discovery.V12) equipped with a GFP filter set. Light intensities were measured at the surface of assay plates using a light meter. For all photostimulation experiments, control and experimental animals were treated similarly except that controls did not receive retinal exposure.

#### uv1 stimulation

Single animals were monitored in the presence of retinal. After the first egg-laying event of an active phase, the animals were immediately exposed to blue light (100 W/m^2^) for 5 seconds and subsequent egg-laying events were monitored. Videotaping was continued for 5–6 mins after the last egg-laying event to ensure the end of an active phase. We noted that photostimulation elicited an escape-like response in a subset of animals expressing the *Pocr-2*::*ChR2*::*ocr-2 3’UTR* transgene (S1 Movie). The *ocr-2* promoter and 3’ UTR used for ChR2 expression in the uv1 cells also drives expression in a small number of sensory neurons near the head [29]. The avoidance behavior we observed likely arises due to activation of these neurons. Notably, this avoidance response also occurred *nlp-7(lf);flp-11(lf)* double mutants where we did not observe appreciable inhibition of egg-laying (S2 Movie), indicating that the egg-laying and locomotory behaviors elicited by light stimulation are separable. NLP-7 and FLP*-11* peptides appear required for inhibition of egg-laying, but are not necessary for the avoidance response to light stimulation.

#### HSN stimulation

On the day of the experiment, single animals were transferred from retinal plates to OP50 plates (without retinal) and assayed immediately for egg-laying response to blue light exposure.

#### Heat shock overexpression experiments

Groups of 10 age-matched adult animals (picked at L4 stage on the day prior to experiments) were transferred to NGM agar plates seeded with OP50, wrapped in parafilm and submerged into a water-bath at 33°C for 30 mins. After 30 mins, the animals were transferred to 20°C and the number of eggs laid were counted every hour.

### Microscopy

#### Expression patterns

Images were acquired using a Zeiss Axioskop 2 microscope system and LSM Pascal 5 imaging software (Zeiss). Images were processed using ImageJ software. Worms were mounted on agarose pads and immobilized with 0.3 M sodium azide. All images were obtained from staged young adult animals (~24hrs after the L4 stage).

#### HSN imaging and quantification

Images were acquired using an Olympus BX51WI spinning disc confocal microscope. Age-matched adults (30 hrs after the L4 stage) were immobilized using 0.3 M sodium azide on slides with 2% agarose pads. z-stack images of HSN synapses and cell bodies were acquired from all animals. Images were analyzed using Volocity6.3 software. Total SNB-1::GFP volume, number of SNB-1::GFP varicosities and GFP::SYD-2 clusters in the HSN synaptic regions were determined as described in [50]. Mean SNB-1::GFP and SYD-2::GFP intensities at HSN synapses were determined by averaging the mean GFP fluorescence intensities from all SNB-1::GFP and SYD-2::GFP varicosities in a single HSN process (within 10 μm of the vulval slit) and subtracting the threshold value (1000 units or 1500 units above mean background fluorescence for SNB-1::GFP or SYD-2::GFP respectively). DsRed2 intensities were measured similarly in the same exact regions occupied by SNB-1::GFP.

SNB-1::GFP and DsRed2 intensities in HSN cell bodies were determined by calculating their mean intensities within each cell body and subtracting the threshold value (1500 units above background).

## Supporting information

S1 Fig *nlp-7* gene structure, mutation and expression. (A) Representative confocal images showing distribution of the dense core vesicle marker IDA-1::GFP (green) in a single uv1 cell. IDA-1::GFP shows a punctate distribution and is enriched near the cell periphery. Scale bar, 2 μm. (B) Fluorescent images of whole animals expressing P*nlp-7*::*nlp-7*::SL2::mCherry. Scale bar, 100 μm. (C) Representative confocal images showing coexpression of P*nlp-7*::SL2::mCherry with P*ida-1*::IDA-1::GFP in the uv1 cells, VC4 and VC5 motor neurons. Scale bar, 5 μm. (D) *nlp-7* gene structure. Solid boxes represent exons. Sequences deleted in *tm2984* allele are indicated. Predicted peptide products are shown to the right of corresponding gene models.
(TIF)

S2 Fig *flp-11* gene structure, mutation and expression. (A) Fluorescent confocal images of whole animals expressing P*flp-11*::GFP. Scale bar, 100 μm. (B) *flp-11* gene structure. Solid boxes represent exons. Sequences deleted in *tm2706* allele are indicated. Predicted peptide products are shown to the right of corresponding gene models.
(TIF)

S3 Fig uv-1-specific expression of *flp-11* reverses constitutive egg-laying in *nlp-7;flp-11* double mutants. Distribution of the developmental stages of eggs laid by either *nlp-7;flp-11* double mutants (upper) or with uv1-specific expression of either *flp-11* (middle) or *nlp-7* (lower) in *nlp-7;flp-11* double mutants. uv1-specific expression of either precursor produced a significant decrease in the proportion of eggs that were laid as 1–8 cell embryos. p<0.0001, Fisher’s exact test.
(TIF)

S4 Fig Comparison of egg-laying rate in M9 versus plates. Quantification of eggs laid in 1 hour for the genotypes indicated, in either control buffer (M9) or on NGM plates seeded with a thin bacterial lawn. Bars represent mean ± SEM for each condition. Numbers above bars indicate n for each condition. Data for M9 were duplicated from Fig 7.
(TIF)

S5 Fig Characterization of synapses and cell bodies of HSNs. (A, B) Quantification of total SNB-1::GFP volume (A) and number of SNB-1::GFP varicosities (B) in HSN synaptic regions for the genotypes indicated. SNB-1::GFP volume greater then 1 μm^3^ was considered as a varicosity [50]. (C) Representative confocal images of HSN cell bodies in transgenic animals expressing the synaptic vesicle marker SNB-1::GFP and DsRed2 in the HSNs (*vsIs103*, *Ptph-1*::SNB-1::GFP; *Ptph-1*::DsRed2) for the genotypes indicated. Scale bar, 3 μm. (D, E) Quantification of average SNB-1::GFP (D) or DsRed2 (E) intensity in cell bodies of the HSNs for the genotypes indicated. Bars represent mean ± SEM for each condition. Numbers in bars indicate the n for each condition. *p<0.05, ANOVA with Sidak’s test.
(TIF)

S6 Fig Localization of active zone marker SYD-2 in HSNs. (A) Representative confocal images of HSN synapses in transgenic animals expressing the active zone marker GFP::SYD-2 in the HSNs (*wyIs12*, *Punc-86*::GFP::SYD-2; *Podr-1*::GFP) for the genotypes indicated. Scale bar, 3 μm. (B, C) Quantification of average GFP::SYD-2 intensity (B) or number of SYD-2 clusters (C) in the HSN synaptic region for the genotypes indicated. Bars represent mean ± SEM for each condition. Numbers in bars indicate the n for each condition.
(TIF)

S7 Fig NLP-7 and FLP-11 shape the timing of egg-laying through negative regulation of serotonergic HSN signaling. (A) During the uv1 “off” phase, spontaneous activity of the HSN neurons elicits serotonin release onto the Vm2 vulval muscles, triggering entry into an active phase of egg-laying. Reduced levels of uv1 activity promote “short” inactive phases (bottom). (B) During the uv1 “on” phase, activation of the uv1 cells triggers release of NLP-7 and FLP-11 peptides. Release of these peptides promotes the termination of an active phase and lengthens the duration of the inactive phase (bottom), at least in part, by reducing serotonergic activation of vulval muscles. Under normal (favorable) conditions, cycles of uv1 activity shape the timing of egg-laying events. Solid lines indicate synaptic connections. Dashed lines indicate volume transmission. Arrows indicate excitation. Circles indicate inhibition. In the lower panel of A and B, each tick mark represents a single egg-laying event.
(TIF)

S1 Movie Egg-laying response to uv1 photostimulation. Light stimulation of the uv1 cells inhibits egg-laying in a control animal expressing *Pocr-2*::*ChR2*::*YFP*::*ocr-2* 3’UTR. Egg-laying events are indicated by numbers. Light stimulation is initiated immediately following the first egg-laying event. The timing and duration of blue light are indicated by blue text. The animal performs an avoidance response upon blue light exposure, likely due to ChR2 expression in 2–3 head sensory neurons (see Methods). Video is played 6X faster than real time.
(MP4)

S2 Movie Egg-laying response to uv1 photostimulation. *nlp-7(lf);flp-11(lf)* double mutant animal expressing channelrhodopsin in the uv1 cells continues egg-laying following light stimulation of the uv1 cells. Note that the avoidance response to light stimulation is unaffected by combined deletion of *nlp-7* and *flp-11*, indicating that NLP-7 and FLP-11 peptides are required for inhibition of the egg-laying response but not for the execution of the avoidance behavior. Egg-laying events are indicated by numbers (black). Light stimulation is initiated immediately following the first egg-laying event. The timing and duration of stimulation are indicated by blue text. Video is played 6X faster than real time.
(MP4)

S3 Movie Egg-laying response to HSN photostimulation. Light stimulation of the HSNs in a control animal expressing *Pegl-6a*::ChR2::YFP. Timing of light stimulation is indicated by white text. The animal initiates a burst of egg-laying a few seconds following the onset of light stimulation.
(MOV)
