# Supplementary figures and images for: Local neuropeptide signaling modulates serotonergic transmission to shape the temporal organization of C. elegans egg-laying behavior
Source: PLoS Genet. 2017 Apr 6;13(4):e1006697. doi: 10.1371/journal.pgen.1006697 (PMC5398689; doi:10.1371/journal.pgen.1006697)

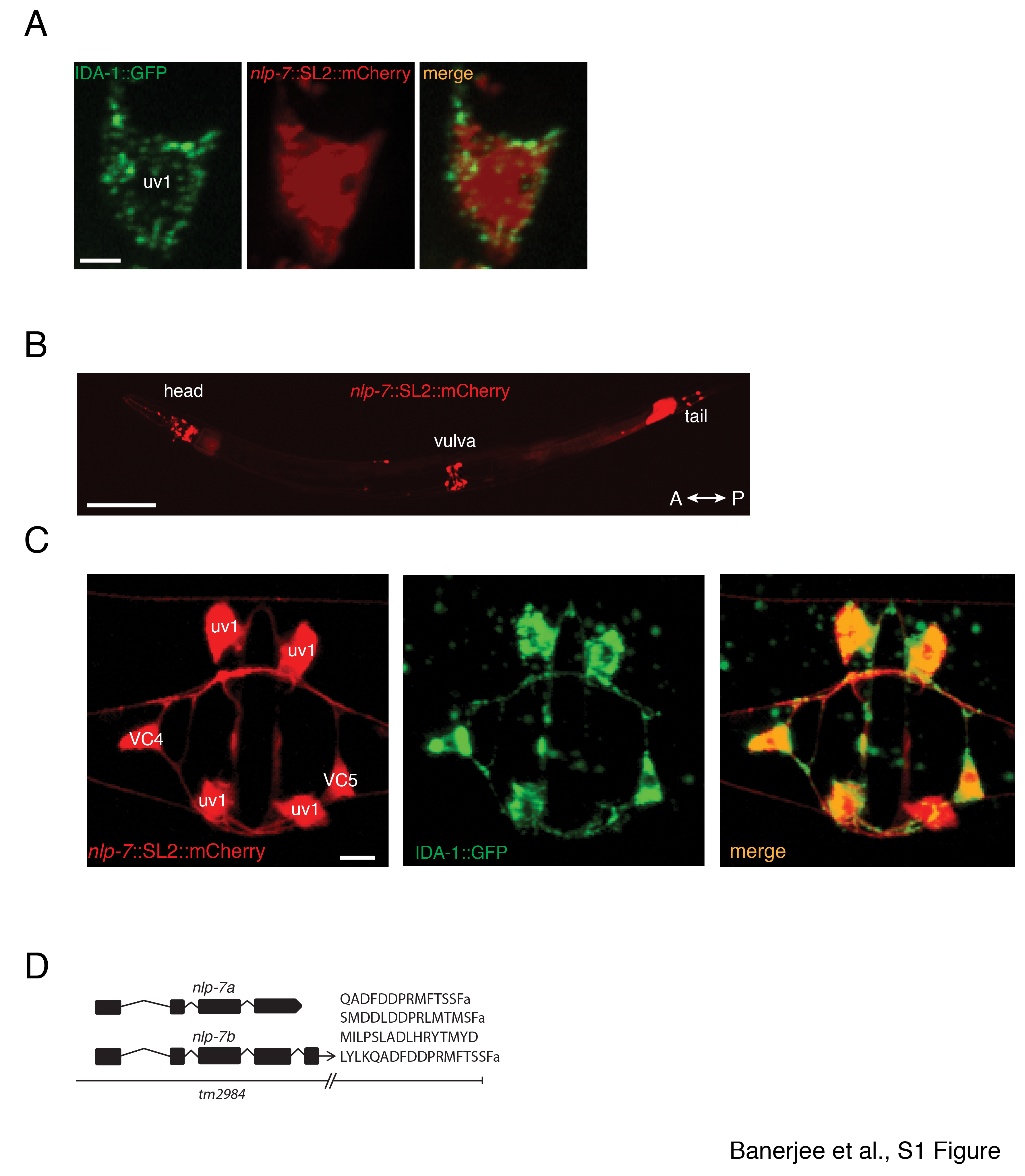

Supplement: S1 Fig — (A) Representative confocal images showing distribution of the dense core vesicle marker IDA-1::GFP (green) in a single uv1 cell. IDA-1::GFP shows a punctate distribution and is enriched near the cell periphery. Scale bar, 2 μm. (B) Fluorescent images of whole animals expressing Pnlp-7::nlp-7::SL2::mCherry. Scale bar, 100 μm. (C) Representative confocal images showing coexpression of Pnlp-7::SL2::mCherry with Pida-1::IDA-1::GFP in the uv1 cells, VC4 and VC5 motor neurons. Scale bar, 5 μm. (D) nlp-7 gene structure. Solid boxes represent exons. Sequences deleted in tm2984 allele are indicated. Predicted peptide products are shown to the right of corresponding gene models. (TIF) [file pgen.1006697.s001.tif]

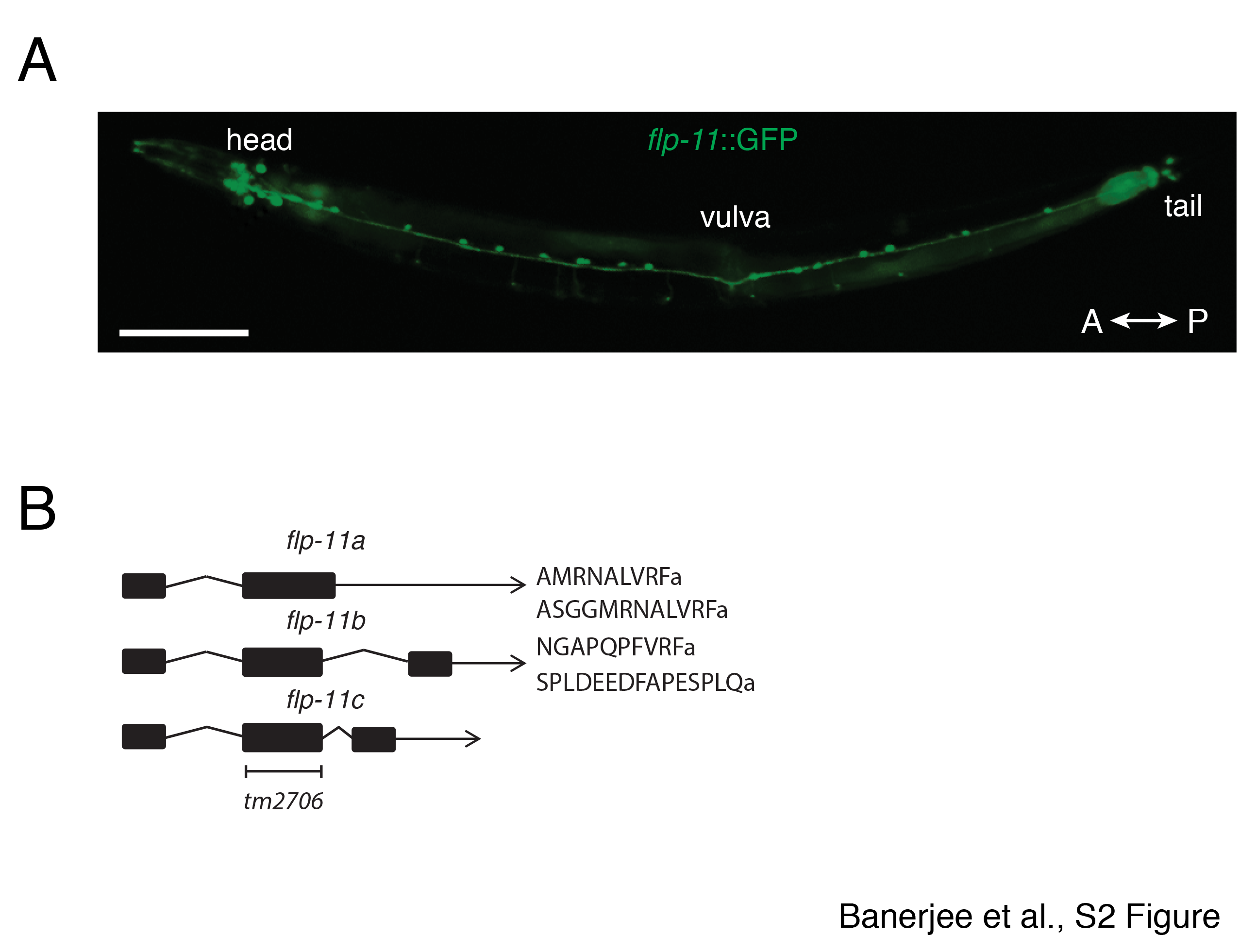

Supplement: S2 Fig — (A) Fluorescent confocal images of whole animals expressing Pflp-11::GFP. Scale bar, 100 μm. (B) flp-11 gene structure. Solid boxes represent exons. Sequences deleted in tm2706 allele are indicated. Predicted peptide products are shown to the right of corresponding gene models. (TIF) [file pgen.1006697.s002.tif]

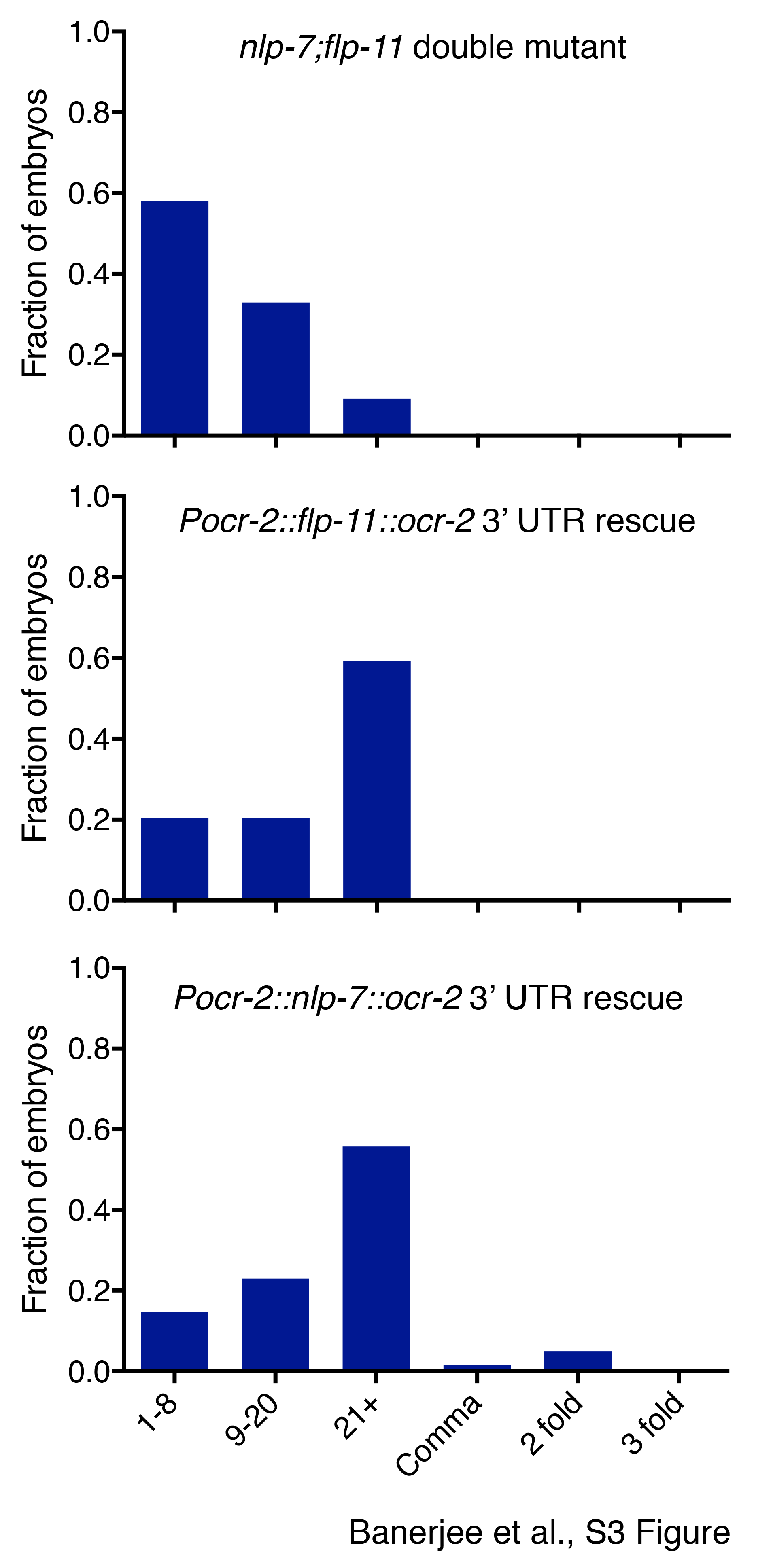

Supplement: S3 Fig — Distribution of the developmental stages of eggs laid by either nlp-7;flp-11 double mutants (upper) or with uv1-specific expression of either flp-11 (middle) or nlp-7 (lower) in nlp-7;flp-11 double mutants. uv1-specific expression of either precursor produced a significant decrease in the proportion of eggs that were laid as 1–8 cell embryos. p<0.0001, Fisher’s exact test. (TIF) [file pgen.1006697.s003.tif]

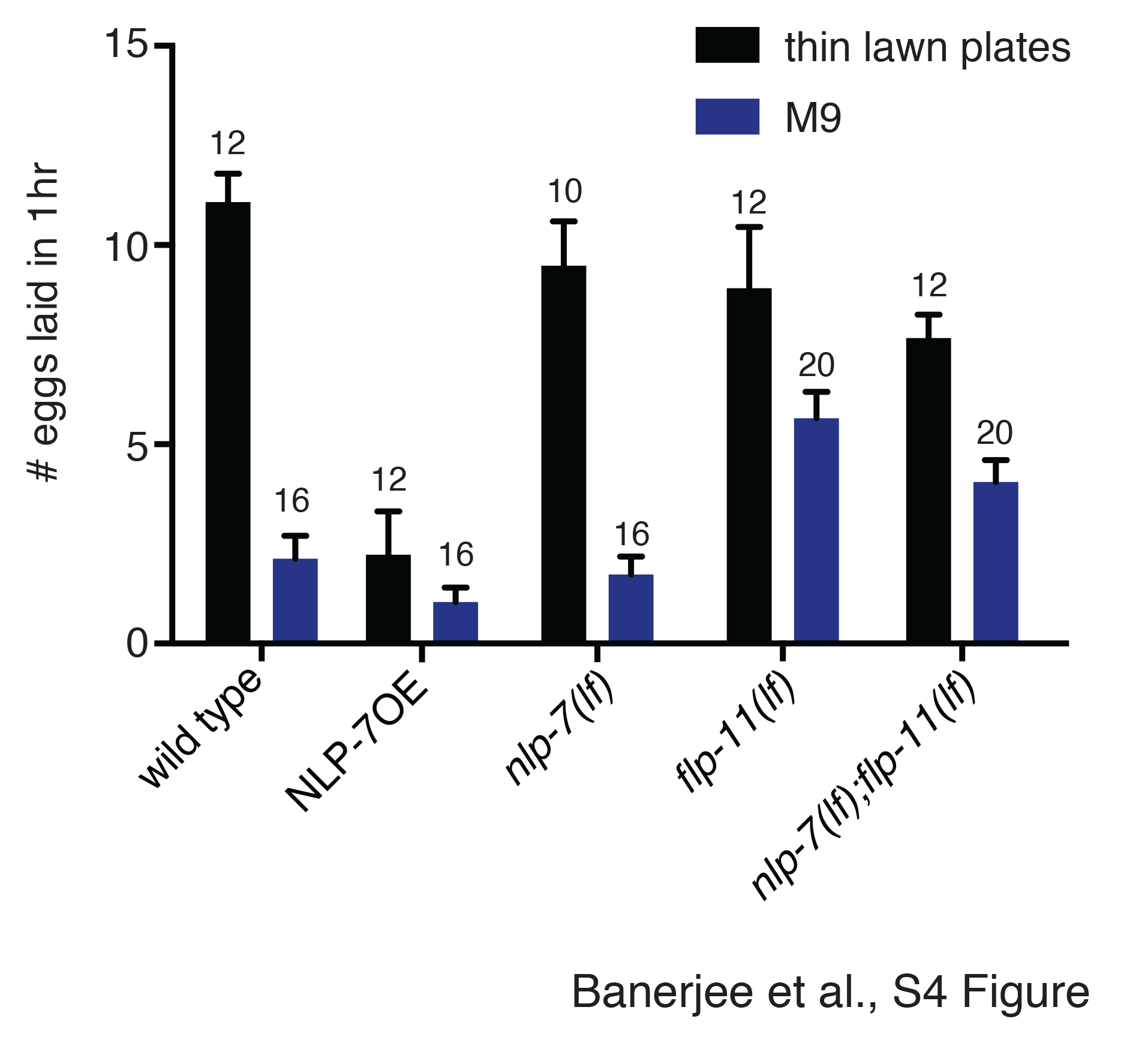

Supplement: S4 Fig — Quantification of eggs laid in 1 hour for the genotypes indicated, in either control buffer (M9) or on NGM plates seeded with a thin bacterial lawn. Bars represent mean ± SEM for each condition. Numbers above bars indicate n for each condition. Data for M9 were duplicated from Fig 7. (TIF) [file pgen.1006697.s004.tif]

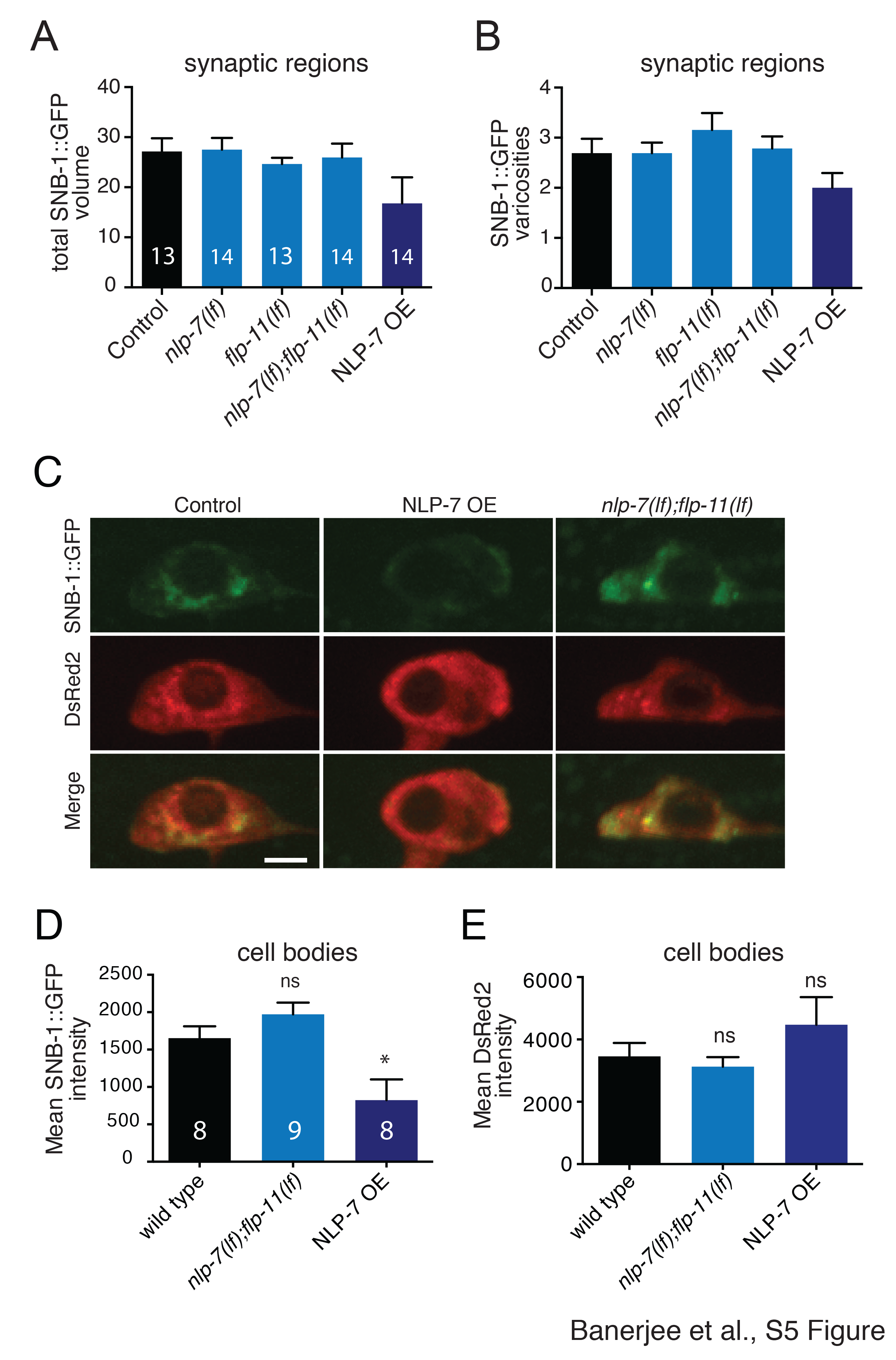

Supplement: S5 Fig — (A, B) Quantification of total SNB-1::GFP volume (A) and number of SNB-1::GFP varicosities (B) in HSN synaptic regions for the genotypes indicated. SNB-1::GFP volume greater then 1 μm3 was considered as a varicosity [50]. (C) Representative confocal images of HSN cell bodies in transgenic animals expressing the synaptic vesicle marker SNB-1::GFP and DsRed2 in the HSNs (vsIs103, Ptph-1::SNB-1::GFP; Ptph-1::DsRed2) for the genotypes indicated. Scale bar, 3 μm. (D, E) Quantification of average SNB-1::GFP (D) or DsRed2 (E) intensity in cell bodies of the HSNs for the genotypes indicated. Bars represent mean ± SEM for each condition. Numbers in bars indicate the n for each condition. *p<0.05, ANOVA with Sidak’s test. (TIF) [file pgen.1006697.s005.tif]

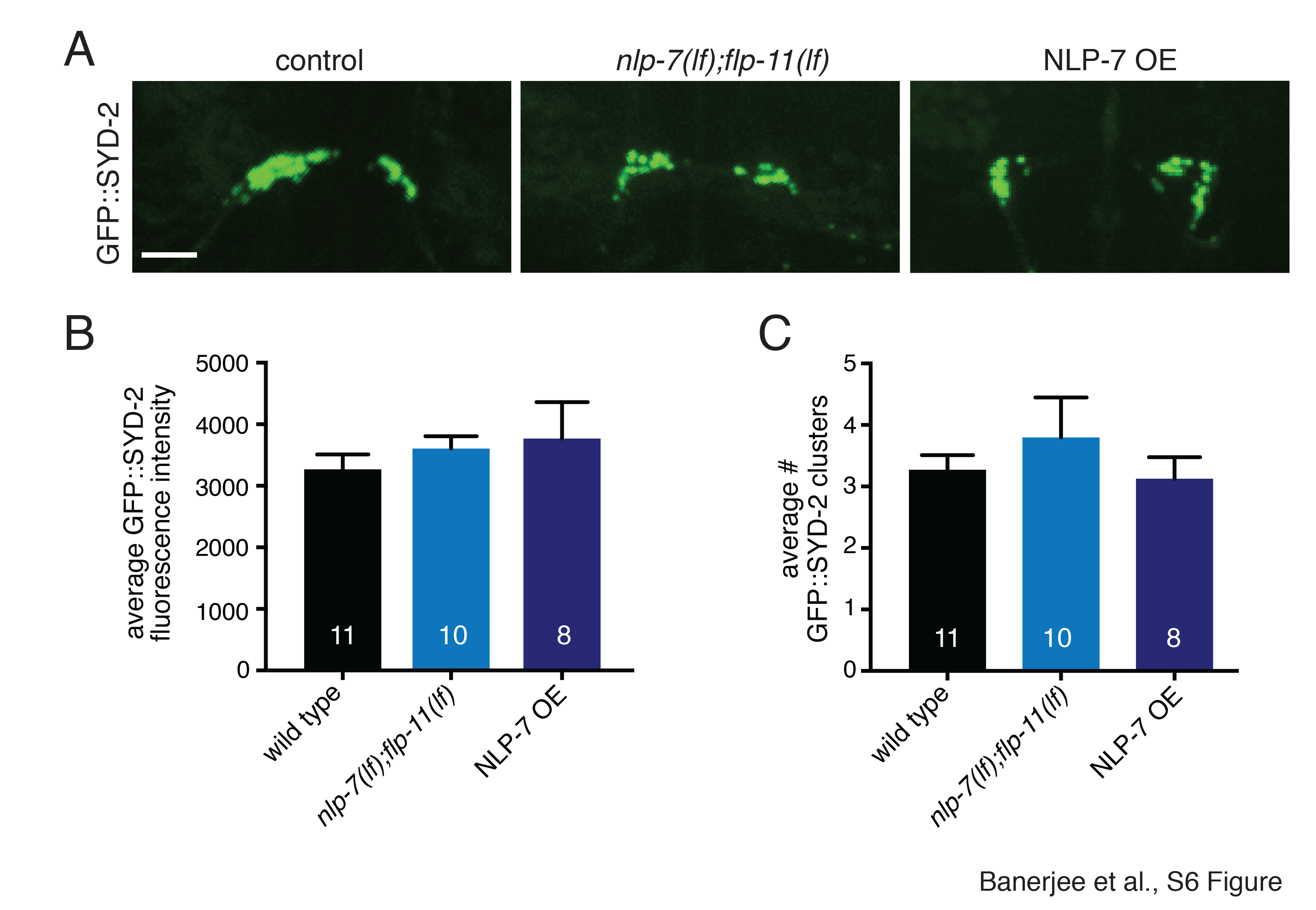

Supplement: S6 Fig — (A) Representative confocal images of HSN synapses in transgenic animals expressing the active zone marker GFP::SYD-2 in the HSNs (wyIs12, Punc-86::GFP::SYD-2; Podr-1::GFP) for the genotypes indicated. Scale bar, 3 μm. (B, C) Quantification of average GFP::SYD-2 intensity (B) or number of SYD-2 clusters (C) in the HSN synaptic region for the genotypes indicated. Bars represent mean ± SEM for each condition. Numbers in bars indicate the n for each condition. (TIF) [file pgen.1006697.s006.tif]

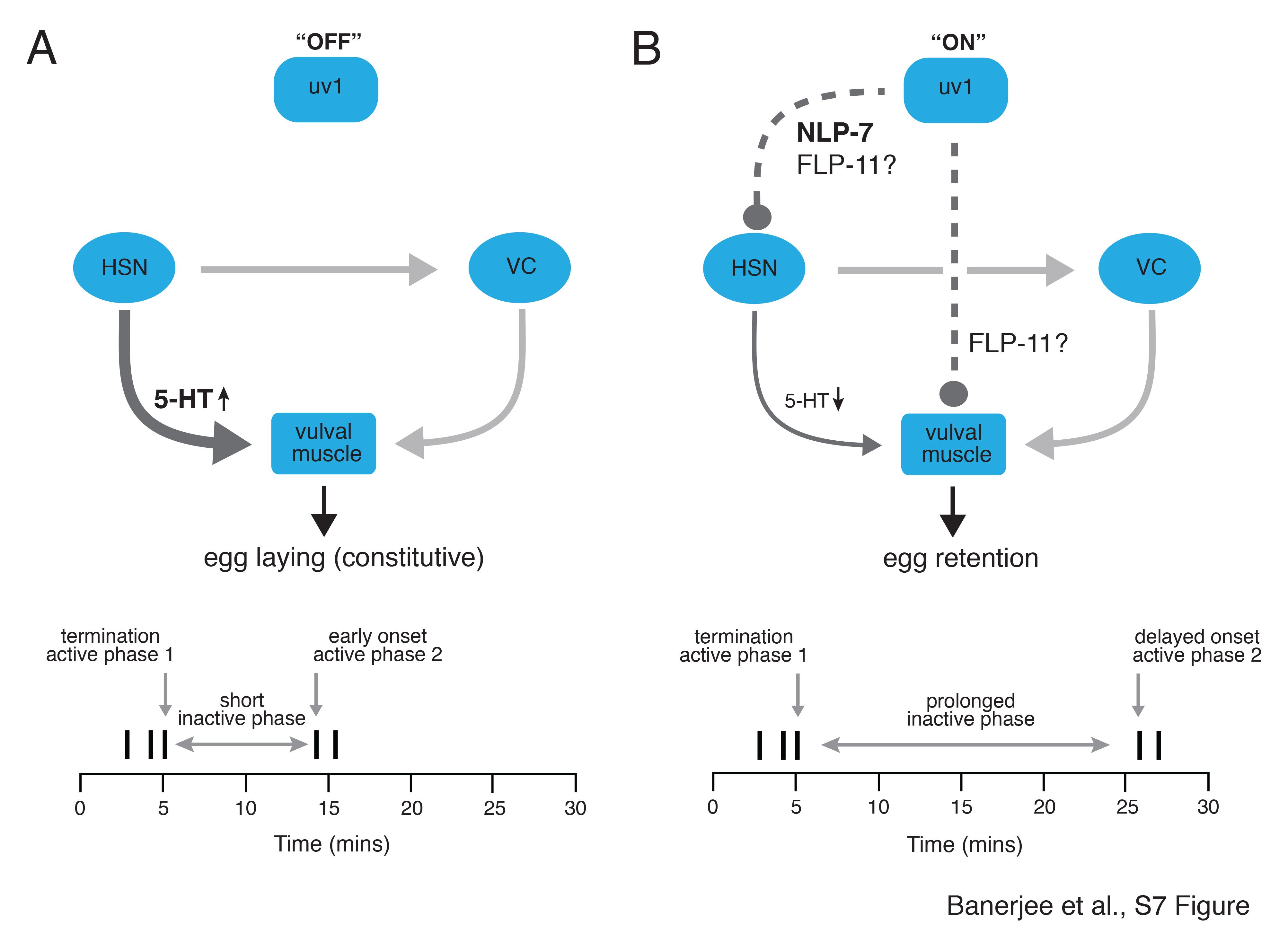

Supplement: S7 Fig — (A) During the uv1 “off” phase, spontaneous activity of the HSN neurons elicits serotonin release onto the Vm2 vulval muscles, triggering entry into an active phase of egg-laying. Reduced levels of uv1 activity promote “short” inactive phases (bottom). (B) During the uv1 “on” phase, activation of the uv1 cells triggers release of NLP-7 and FLP-11 peptides. Release of these peptides promotes the termination of an active phase and lengthens the duration of the inactive phase (bottom), at least in part, by reducing serotonergic activation of vulval muscles. Under normal (favorable) conditions, cycles of uv1 activity shape the timing of egg-laying events. Solid lines indicate synaptic connections. Dashed lines indicate volume transmission. Arrows indicate excitation. Circles indicate inhibition. In the lower panel of A and B, each tick mark represents a single egg-laying event. (TIF) [file pgen.1006697.s007.tif]
